# Trends in traumatic brain injury mortality in China, 2006–2013: A population-based longitudinal study

**DOI:** 10.1371/journal.pmed.1002332

**Published:** 2017-07-11

**Authors:** Peixia Cheng, Peng Yin, Peishan Ning, Lijun Wang, Xunjie Cheng, Yunning Liu, David C. Schwebel, Jiangmei Liu, Jinlei Qi, Guoqing Hu, Maigeng Zhou

**Affiliations:** 1 Department of Epidemiology and Health Statistics, Xiangya School of Public Health, Central South University, Changsha, China; 2 National Center for Chronic and Noncommunicable Disease Control and Prevention, Chinese Center for Disease Control and Prevention, Beijing, China; 3 Department of Psychology, University of Alabama at Birmingham, Birmingham, Alabama, United States of America; Oregon Health and Science University, UNITED STATES

## Abstract

**Background:**

Traumatic brain injury (TBI) is a significant global public health problem, but has received minimal attention from researchers and policy-makers in low- and middle-income countries (LMICs). Epidemiological evidence of TBI morbidity and mortality is absent at the national level for most LMICs, including China. Using data from China’s Disease Surveillance Points (DSPs) system, we conducted a population-based longitudinal analysis to examine TBI mortality, and mortality differences by sex, age group, location (urban/rural), and external cause of injury, from 1 January 2006 to 31 December 2013 in China.

**Method and findings:**

Mortality data came from the national DSPs system of China, which has coded deaths using the International Classification of Diseases–10th Revision (ICD-10) since 2004. Crude and age-standardized mortality with 95% CIs were estimated using the census population in 2010 as a reference population. The Cochran–Armitage trend test was used to examine the significance of trends in mortality from 2006 to 2013. Negative binomial models were used to examine the associations of TBI mortality with location, sex, and age group. Subgroup analysis was performed by external cause of TBI. We found the following: (1) Age-adjusted TBI mortality increased from 13.23 per 100,000 population in 2006 to 17.06 per 100,000 population in 2008 and then began to fall slightly. In 2013, age-adjusted TBI mortality was 12.99 per 100,000 population (SE = 0.13). (2) Compared to females and urban residents, males and rural residents had higher TBI mortality risk, with adjusted mortality rate ratios of 2.57 and 1.71, respectively. TBI mortality increased substantially with older age. (3) Motor vehicle crashes and falls were the 2 leading causes of TBI mortality between 2006 and 2013. TBI deaths from motor vehicle crashes in children aged 0–14 years and adults aged 65 years and older were most often in pedestrians, and motorcyclists were the first or second leading category of road user for the other age groups. (4) TBI mortality attributed to motor vehicle crashes increased for pedestrians and motorcyclists in all 7 age groups from 2006 to 2013. Our analysis was limited by the availability and quality of data in the DSPs dataset, including lack of injury-related socio-economic factors, policy factors, and individual and behavioral factors. The dataset also may be incomplete in TBI death recording or contain misclassification of mortality data.

**Conclusions:**

TBI constitutes a serious public health threat in China. Further studies should explore the reasons for the particularly high risk of TBI mortality among particular populations, as well as the reasons for recent increases in certain subgroups, and should develop solutions to address these challenges. Interventions proven to work in other cultures should be introduced and implemented nationwide. Examples of these in the domain of motor vehicle crashes include policy change and enforcement of laws concerning helmet use for motorcyclists and bicyclists, car seat and booster seat use for child motor vehicle passengers, speed limit and drunk driving laws, and alcohol ignition interlock use. Examples to prevent falls, especially among elderly individuals, include exercise programs, home modification to reduce fall risk, and multifaceted interventions to prevent falls in all age groups.

## Abstract 2

In a population-based cohort study, Guoqing Hu and colleagues examine trends in traumatic brain injury mortality in China, 2006–2013.

## Author summary

**Why was this study done?:**

- Traumatic brain injury (TBI) is a serious public health problem worldwide, but epidemiological evidence is scarce in low- and middle-income countries like China.
- Population-based mortality from TBI remains unreported at the national level for China.

**What did the researchers do and find?:**

- Using data from the national Disease Surveillance Points (DSPs) system, we conducted a population-based longitudinal analysis to report trends in TBI mortality and differences based on location (urban/rural), sex, age group, and external cause of TBI from 2006 through 2013 in China.
- Overall TBI-related mortality remained relatively stable and at a high level, ranging from 12.99 to 17.06 per 100,000 population, between 2006 and 2013 in China.
- Males, rural residents, and older adults consistently had significantly higher TBI mortality compared to females, urban residents, children, and young adults in China from 2006 to 2013.
- Motor vehicle crashes and falls were the 2 most important causes of TBI mortality for Chinese individuals younger than 75 years, but falls were the leading cause of TBI deaths for adults aged 75 years and older between 2006 and 2013.
- TBI deaths attributed to motor vehicle crashes in children aged 0–14 years and adults aged 65 years and older were most often in pedestrians, but motorcyclists were as vulnerable to TBI deaths as pedestrians among Chinese residents aged 15–64 years.

**What do these findings mean?:**

- TBI represents a serious public health problem and should be considered a public health priority in China.
- Proven and affordable interventions should be introduced in China and implemented nationwide to protect citizens—and especially high-risk populations such as males, rural residents, and older adults—from TBI injuries and deaths.

## Introduction

Traumatic brain injury (TBI) is defined as an alteration of brain function or evidence of brain pathology caused by an external force [1]. It is a serious public health problem worldwide. Each year, TBI causes a substantial number of deaths and temporary and permanent disabilities [2–5]. In the United States, for example, a total of approximately 2.5 million emergency department visits, 282,000 hospitalizations, and 56,000 deaths occurred due to TBI in 2013 [6]. In Europe, Majdan et al. [7] report that TBI caused about 82,000 deaths and was recorded in 2.1 million hospital discharges in 2012.

Understanding the patterns, causes, and trends of TBI is critical for quantifying the severity of the problem, identifying risk factors, developing prevention solutions, and evaluating interventions and policies at local, national, and global levels. Unfortunately, few publications have addressed the epidemiological characteristics of TBI morbidity and mortality, and most available data are from high-income countries (HICs) [8–12]. Rigorous national epidemiological studies are particularly scarce for TBI in most low- and middle-income countries (LMICs). For China, the only available epidemiological studies on TBI that have been published in international or domestic academic journals were conducted among outpatients and/or inpatients at local hospitals, and the results cannot be generalized to the national population [13–15].

The absence of high-quality research evidence from LMICs like China not only prevents the public and policy-makers from recognizing the scope, severity, and urgency of TBI, and therefore taking actions to curb the threat, but also substantially undermines the reliability and validity of the estimation of global TBI burden, which depends greatly on the availability and quality of research evidence from LMICs at the national and local levels [16,17].

To address these research gaps, we used mortality data from the nationally representative Disease Surveillance Points (DSPs) system in China to investigate TBI mortality and differences in mortality based on location (urban/rural), sex, age group, and external cause of TBI from 2006 to 2013 in China.

## Methods

### Ethics concerns

This secondary analysis was approved by the ethics committee of Xiangya School of Public Health, Central South University. Data were de-identified, and informed consent was not required.

### Design

We conducted a population-based longitudinal analysis to examine trends in TBI mortality and differences based on location (urban/rural), sex, age group, and external cause of TBI from 1 January 2006 through 31 December 2013. RECORD guidelines (S1 RECORD Checklist) were adhered to in reporting this study.

This study was part of a larger study focusing on the fatality burden from several major causes of injury, including TBI, in China using the DSPs data. Ideas were conceived in August 2016, and a research plan was prepared, listing the key points of secondary data analysis, including research questions, study population, data sources, definitions of primary injuries, cause classifications, related International Classification of Diseases–10th Revision (ICD-10) codes, primary outcome measures, explanatory factors (location, sex, age group, and year), statistical analyses, and ethical reviews (S1 Protocol). In mid-October 2016, we completed the extraction and collation of data from the DSPs system. We then analyzed the data using the preexisting data analysis plan.

### Data sources

Mortality rates were derived from the DSPs dataset. Mortality data for this study can be accessed by contacting the Division of Vital Registration and Cause of Death Surveillance, National Center for Chronic and Noncommunicable Disease Control and Prevention, Chinese Center for Disease Control and Prevention (CDC) (contact email: crvsdata@chinacdc.cn). The DSPs system was first established by the Chinese central government in 1978, and data are collected under the direction of the China CDC [18]. Each surveillance point represents a single district (urban area) or county (rural area), and all residents of that geographic area are covered by the DSPs system [19]. In 2004–2006, an important improvement was made, raising the number of surveillance points to 161 (64 urban, 97 rural) across all 31 Chinese provinces and creating a dataset that is demographically representative of the full country [19–21]. Starting in 2008, the Chinese government introduced a web-based approach to report death cases using the DSPs system [22,23], and the timeliness of data reporting has improved remarkably. In 2013, the number of surveillance points was increased to 605 points [24], but we used data only from the 161 points available beginning in 2006 for this analysis to avoid potential impact on our research from the DSPs update in 2013.

All causes of death in DSPs are determined according to a standard protocol by trained coders located in hospitals or local CDC offices. Since 2004, coding has been conducted using ICD-10 codes. Duplicate deaths are identified and eliminated as part of routine processing operations at the provincial and county levels. An internal procedural check system evaluates timeliness, completeness, and accuracy of data reporting, and statistical measures (e.g., the standard United Nations Age–Sex Accuracy Index) are employed to monitor data quality [18]. Further, a fixed national sample survey is conducted at all DSPs locations every 3 years to obtain extra information that permits adjustment for underreporting [25]. Previous studies indicate that the DSPs data are generally reliable, valid, and nationally representative [18,25].

### Definition of traumatic brain injury

TBI-induced deaths were identified according to the ICD-10 codes recommended by the US CDC [26,27], based on the following diagnosis codes: S01.0–S01.9, S02.0, S02.1, S02.3, S02.7–S02.9, S04.0, S06.0–S06.9, S07.0, S07.1, S07.8, S07.9, S09.7−S09.9, T01.0, T02.0, T04.0, T06.0, T90.1, T90.2, T90.4, T90.5, T90.8, and T90.9. Based on published studies and ICD-10 codes of external cause of injury [8,28], we classified the underlying external causes of TBI deaths into 4 categories: motor vehicle crashes (V30–V79 [.4–.9], V81.1, V82.1, V83–V86 [.0–.3], V20–V28 [.3–.9], V29 [.4–.9], V12–V14 [.3–.9], V19 [.4–.6], V02–V04 [.1, .9], V09.2, V80 [.3–.5], V87 [.0–.8], V89.2, X82, Y03, Y32), falls (W00–W19, X80, Y01, Y30), struck by and against (W20–W22, W50–W52, X79, Y00, Y04, Y29, Y35.3), and all others (remaining ICD-10 codes for all other causes). To detail the subgroups of TBI deaths from motor vehicle crashes, we report the results by road user category. Road users were classified into 5 categories according to the ICD-10 injury matrix recommended by the US CDC [29]: vehicle occupant (V30−V79 [.4−.9], V81.1, V82.1, V83−V86 [.0−.3]), motorcyclist (V20−V28 [.3−.9], V29 [.4−.9]), pedal cyclist (V12−V14 [.3−.9], V19 [.4−.6]), pedestrian (V02−V04 [.1, .9], V09.2), and all others (V80 [.3−.5], V87 [.0−.8], V89.2).

### Statistical analyses

The DSPs database underwent several changes in its sampling methods during the 1990s, mid-2000s, and recently. To eliminate bias from these changes, we analyzed mortality data from the same 161 surveillance points from 2006 to 2013, in this study. We calculated the overall and cause-specific age-standardized mortality over that time period. The direct standardization method was adopted to adjust for the impact of the population structure. The census population in 2010 was used as the reference population. The 95% confidence intervals of mortality rates were calculated.

Because substantial age variations in TBI morbidity and mortality have been reported in previous publications [8,26], we created 7 age groups: 0–4 years, 5–14 years, 15–24 years, 25–44 years, 45–64 years, 65–74 years, and 75 years and older. We calculated sex-, age-, location-, and cause-specific death rates to inform policy-making priorities. Pearson chi-square tests were used to test differences in mortality rates between subgroups. When the assumption for the chi-square test was violated, Fisher’s exact test was used.

The Cochran–Armitage trend test was used to examine the significance of trends in mortality. Subgroup analysis was conducted by strata of location (urban/rural), sex, age group, and external cause of TBI. Because of the overdispersion of TBI-induced deaths, we used negative binomial models to explore the associations of deaths with socio-demographic factors including location (urban/rural), sex, age group, and year [30,31]. Mortality rate ratios (MRRs) and corresponding 95% CIs quantified the extent of associations. Following strategies in a previous report [32], we tested the significance of the interaction of age group × year by comparing negative binomial models with and without multiplicative interaction terms using the likelihood ratio test.

All statistical analyses were conducted using Stata statistical software version 12.1. Differences were considered statistically significant in 2-tailed tests if *p-*values were less than 0.05.

## Results

### Surveillance sample characteristics of disease surveillance points

Between 2006 and 2013, the population covered by the 161 included surveillance points in the DSPs database increased from 76 million to 83 million, accounting, respectively, for 5.8% and 6.3% of the total population of China (S1 Table). Across the study time period, males generally represented 50.3%–51.1% of the surveillance sample, the proportion of urban residents rose from 37.4% to 43.5%, and older age groups represented a gradually growing portion of the whole population. All of these trends are consistent with national demographical statistics in China over the same years.

### Overall traumatic-brain-injury-induced mortality and trends

In total, 93,793 TBI-induced deaths were captured by the DSPs between 1 January 2006 and 31 December 2013. Age-adjusted TBI mortality first increased from 13.23 per 100,000 population in 2006 to 17.06 per 100,000 population in 2008, and then started to fall gradually. In 2013, age-adjusted TBI mortality was 12.99 per 100,000 population (SE = 0.13) (Table 1). After adjusting for location (urban/rural), sex, and age group, TBI mortality from 2007 to 2011 was increased, respectively, by 17%, 31%, 15%, 27%, and 19% compared with 2006, and then remained at a similar level from 2012 to 2013 (Table 2).

**Table 1:** Mortality rates of traumatic brain injury per 100,000 population by location, sex, age group, and external cause of injury in China, 2006–2013.

Variable	Total		2006		2007		2008		2009		2010		2011		2012		2013		Trend χ^2^^#^	*p-*Value
	Rate^†^	SE	Rate^†^	SE	Rate^†^	SE	Rate^†^	SE	Rate^†^	SE	Rate^†^	SE	Rate^†^	SE	Rate^†^	SE	Rate^†^	SE		
**Total**	14.97	0.05	13.23	0.13	15.38	0.14	17.06	0.15	15.22	0.14	16.13	0.14	15.27	0.14	14.46	0.13	12.99	0.13	42.78	<0.001
**Location**																				
Urban	9.92	0.06	9.06	0.18	10.05	0.19	12.23	0.20	11.22	0.20	11.27	0.20	9.54	0.17	9.08	0.16	7.94	0.15	78.45	<0.001
Rural	18.55	0.07	15.90	0.18	18.82	0.20	20.26	0.20	17.89	0.19	19.37	0.20	20.10	0.21	18.78	0.20	17.11	0.19	13.15	<0.001
**Sex**																				
Male	22.64	0.08	20.23	0.23	23.19	0.24	25.74	0.26	23.02	0.24	24.23	0.25	23.31	0.24	21.92	0.23	19.38	0.21	42.70	<0.001
Female	7.21	0.05	6.30	0.13	7.43	0.14	8.22	0.15	7.29	0.14	7.91	0.14	7.20	0.13	6.94	0.13	6.53	0.13	4.21	0.040
**Age group (years)**																				
0–4	4.34	0.11	3.86	0.29	4.14	0.30	4.29	0.30	3.80	0.28	4.28	0.30	5.01	0.33	4.73	0.33	4.71	0.32	8.35	0.004
5–14	2.83	0.06	2.47	0.16	3.12	0.18	3.39	0.19	2.84	0.18	3.10	0.19	2.92	0.19	2.51	0.17	2.23	0.16	5.74	0.017
15–24	8.54	0.09	7.54	0.24	9.47	0.27	9.84	0.27	8.91	0.26	9.59	0.27	9.01	0.26	7.20	0.23	6.76	0.23	31.61	<0.001
25–44	14.43	0.08	13.97	0.23	15.84	0.25	17.28	0.26	15.19	0.24	15.35	0.25	13.89	0.23	12.76	0.22	11.23	0.21	216.28	<0.001
45–64	19.79	0.11	15.74	0.30	18.56	0.33	22.09	0.35	19.50	0.32	21.55	0.33	21.11	0.32	20.98	0.31	18.34	0.28	36.09	<0.001
65–74	26.96	0.28	23.23	0.75	25.84	0.78	28.45	0.81	25.73	0.77	29.37	0.82	26.67	0.74	28.86	0.79	27.23	0.76	15.61	<0.001
≥75	55.48	0.54	50.97	1.60	59.35	1.71	62.29	1.72	58.75	1.63	58.13	1.60	57.14	1.49	52.36	1.33	48.60	1.25	18.18	<0.001
**External cause**																				
MVC	7.01	0.03	5.07	0.08	6.39	0.09	7.00	0.10	6.98	0.09	8.29	0.10	8.02	0.10	7.53	0.10	6.49	0.09	253.88	<0.001
Falls	3.36	0.02	2.93	0.06	3.29	0.07	3.71	0.07	3.39	0.07	3.48	0.07	3.43	0.06	3.35	0.06	3.32	0.06	7.08	0.008
Struck by/against	0.68	0.01	0.69	0.03	0.71	0.03	0.89	0.03	0.67	0.03	0.72	0.03	0.61	0.03	0.61	0.03	0.55	0.03	38.44	<0.001
All others	3.92	0.02	4.54	0.03	5.00	0.08	5.45	0.08	4.18	0.07	3.64	0.07	3.22	0.06	2.97	0.06	2.63	0.06	1,148.73	<0.001

†Mortality rates for total and subgroup traumatic brain injury (except for the 7 age groups) were age-standardized based on the population of China in 2010.

#Mortality trends from 2006 to 2013 were examined using the Cochran–Armitage trend test.

MVC, motor vehicle crash.

**Table 2:** Associations of traumatic brain injury mortality with socio-demographic variables from multivariate negative binomial regression (China, 2006–2013).

Socio-demographic variable	Total TBI mortality			TBI mortality by external cause of injury											
				Motor vehicle crash			Falls			Struck by/against			All others		
	MRR	95% CI bounds		MRR	95% CI bounds		MRR	95% CI bounds		MRR	95% CI bounds		MRR	95% CI bounds	
		Lower	Upper		Lower	Upper		Lower	Upper		Lower	Upper		Lower	Upper
**Age group (reference = 25–44 years)**															
0–4 years	0.37*	0.33	0.43	0.30*	0.26	0.34	0.61*	0.51	0.72	0.23*	0.17	0.32	0.34*	0.29	0.39
5–14 years	0.23*	0.20	0.25	0.22*	0.19	0.25	0.26*	0.22	0.31	0.15*	0.11	0.20	0.21*	0.18	0.24
15–24 years	0.60*	0.53	0.67	0.65*	0.58	0.72	0.48*	0.42	0.56	0.46*	0.38	0.56	0.61*	0.55	0.68
45–64 years	1.46*	1.31	1.64	1.42*	1.28	1.57	1.86*	1.62	2.14	1.18	1.00	1.40	1.41*	1.27	1.57
65–74 years	2.21*	1.97	2.48	1.95*	1.75	2.17	3.94*	3.41	4.54	0.99	0.80	1.23	1.95*	1.74	2.19
75 years and older	5.16*	4.60	5.78	2.62*	2.35	2.92	18.88*	16.41	21.72	1.52*	1.21	1.92	2.89*	2.57	3.25
**Location (reference = urban)**															
Rural	1.71*	1.60	1.82	1.88*	1.77	2.00	1.50*	1.38	1.63	1.93*	1.70	2.19	1.65*	1.54	1.76
**Sex (reference = female)**															
Male	2.57*	2.41	2.74	2.63*	2.46	2.80	2.66*	2.44	2.90	5.00*	4.34	5.77	2.86*	2.67	3.07
**Year (reference = 2006)**															
2007	1.17*	1.03	1.32	1.30*	1.14	1.48	1.11	0.94	1.31	1.07	0.84	1.36	1.07	0.94	1.22
2008	1.31*	1.16	1.49	1.40*	1.23	1.59	1.24*	1.05	1.47	1.34*	1.05	1.70	1.25*	1.10	1.42
2009	1.15*	1.01	1.31	1.38*	1.22	1.57	1.11	0.94	1.32	1.07	0.84	1.36	0.95	0.83	1.08
2010	1.27*	1.12	1.44	1.67*	1.48	1.90	1.20*	1.02	1.42	1.03	0.80	1.31	0.85*	0.74	0.97
2011	1.19*	1.04	1.35	1.65*	1.46	1.87	1.15	0.98	1.36	0.96	0.75	1.23	0.73*	0.64	0.84
2012	1.10	0.97	1.25	1.52*	1.34	1.73	1.15	0.97	1.36	0.92	0.72	1.18	0.66*	0.58	0.76
2013	1.02	0.89	1.15	1.30*	1.15	1.48	1.17	0.99	1.39	0.86	0.67	1.10	0.61*	0.53	0.70

*p < 0.05.

MRR, mortality rate ratio; TBI, traumatic brain injury.

### Urban–rural difference

**Fig 1:**
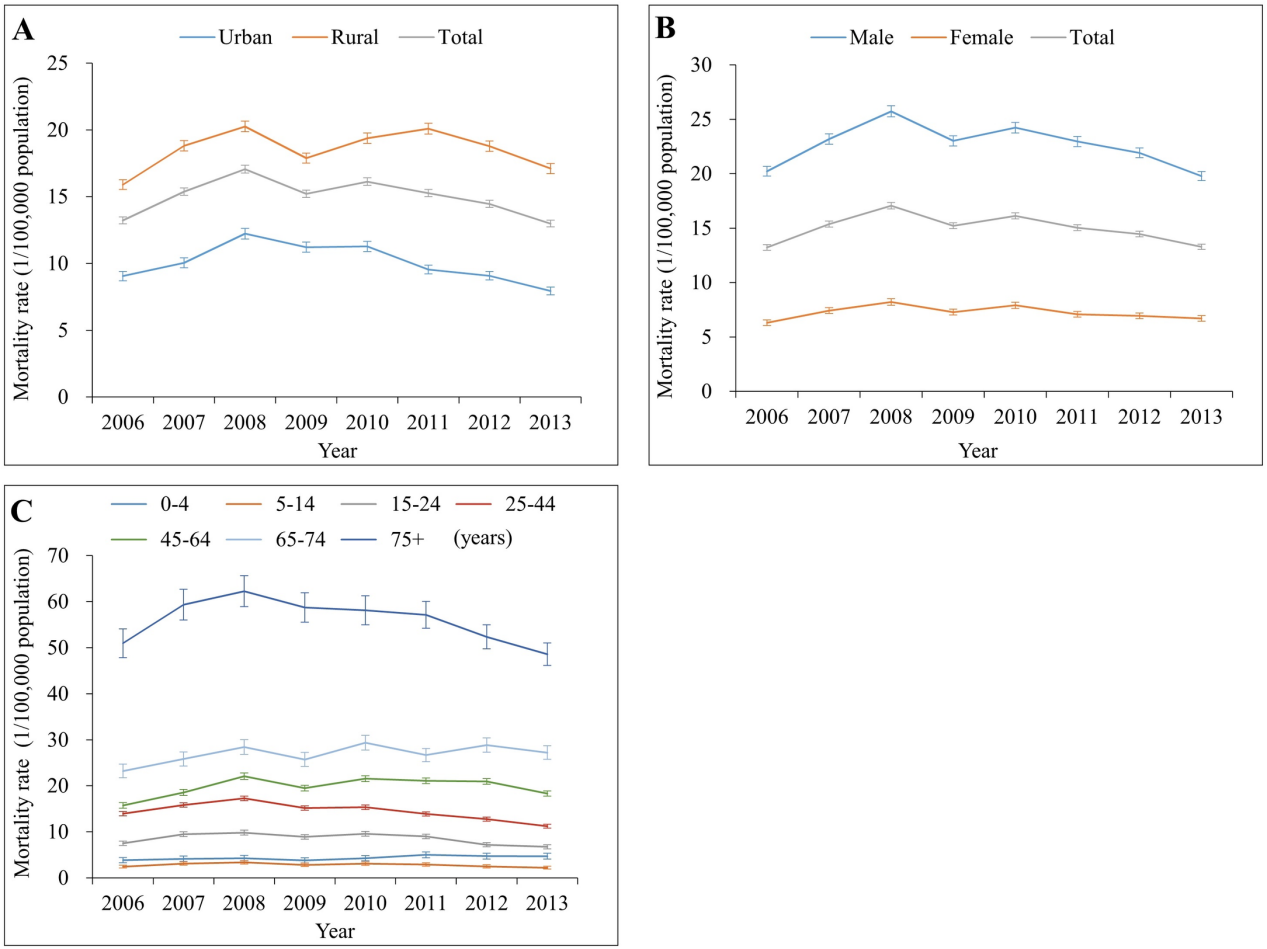
Mortality rates from traumatic brain injury by location (urban/rural), sex, and age group in China, 2006–2013. (A) By location; (B) by sex; (C) by age group. Mortality rates in (A) and (B) were age-standardized based on the population of China in 2010.

Rural residents had much higher TBI mortality rates between 2006 and 2013 than urban residents (age-standardized mortality ratio ranging from 1.6 to 2.2) (S2 Table; Fig 1A). After adjusting for sex, age group, and year, rural residents were at greater risk of dying from TBI than urban residents (MRR for overall TBI: 1.71, 95% CI 1.60–1.82; for motor vehicle crashes: 1.88, 95% CI 1.77–2.00; for falls: 1.50, 95% CI 1.38–1.63; for struck by/against: 1.93, 95% CI 1.70–2.19; Table 2). TBI mortality rates followed similar patterns for both urban and rural residents from 2006 to 2013 (Fig 1A).

### Sex difference

Compared to females, males had about a 3-fold greater risk of dying from TBI during 2006–2013 (S3 Table; Fig 1B). TBI mortality for both males and females showed a similar pattern of change over time in the study period. After adjusting for location (urban/rural), age group, and year, males had 2.57 times the risk of dying from TBI compared to females. The adjusted male–female MRR was 2.63 for TBI from motor vehicle crashes and 2.66 for TBI from falls, but was up to 5.00 for TBI from struck by/against (Table 2).

### Variations across age groups

Large TBI mortality variations were observed between age groups for all years (Fig 1C). For persons aged 5 years and older, TBI mortality increased substantially as age increased. In 2013, children aged 5–14 years had the lowest mortality (2.33 per 100,000 population), while adults aged 75 years and older had the highest mortality (48.60 per 100,000 population) (Table 1); the overall TBI-induced mortality rates by 5-year age group and year are listed in S4 Table.

Trends in TBI mortality between 2006 and 2013 varied with age group. All 7 age groups showed a rising tendency for TBI deaths from 2006 to 2008, but 3 age groups (15–24 years, 25–44 years, and ≥75 years) showed a gradual and substantial decrease in TBI mortality after 2008. The age group 0–4 years showed a slight increase, and the other 3 groups remained nearly unchanged, between 2008 and 2013 (Fig 1C).

Similar and large TBI mortality variations remained between age groups after adjusting for location (urban/rural), sex, and year, but the pattern was different for external cause of TBI (Tables 2 and 3). For example, the adjusted MRR for adults aged 75 years and older compared to adults aged 25–44 years old was 18.88 for falls (95% CI 16.41–21.72) but was far lower than 10 for all other external causes.

**Table 3:** Associations of traumatic brain injury mortality from motor vehicle crashes with socio-demographic variables from multivariate negative binomial regression (China, 2006–2013).

Socio-demographic variable	TBI mortality from motor vehicle crashes by road user category														
	Vehicle occupant			Motorcyclist			Pedal cyclist			Pedestrian			All others		
	MRR	95% CI bounds		MRR	95% CI bounds		MRR	95% CI bounds		MRR	95% CI bounds		MRR	95% CI bounds	
		Lower	Upper		Lower	Upper		Lower	Upper		Lower	Upper		Lower	Upper
**Age group (reference = 25–44 years)**															
0–4 years	0.23*	0.18	0.29	0.05*	0.04	0.07	0.14*	0.09	0.22	0.51*	0.44	0.58	0.27*	0.20	0.38
5–14 years	0.13*	0.10	0.17	0.07*	0.06	0.08	0.29*	0.22	0.37	0.33*	0.29	0.38	0.23*	0.17	0.30
15–24 years	0.49*	0.42	0.57	0.74*	0.67	0.82	0.55*	0.45	0.68	0.60*	0.54	0.67	0.74*	0.61	0.89
45–64 years	1.03	0.90	1.18	0.92	0.84	1.02	2.60*	2.21	3.05	1.79*	1.63	1.96	1.28*	1.08	1.52
65–74 years	0.90	0.76	1.06	0.51*	0.45	0.59	3.59*	3.00	4.30	3.28*	2.97	3.61	1.68*	1.37	2.04
75 years and older	0.89	0.74	1.09	0.36*	0.30	0.43	3.44*	2.81	4.20	5.16*	4.68	5.70	2.05*	1.65	2.55
**Location (reference = urban)**															
Rural	1.97*	1.78	2.19	3.16*	2.92	3.42	1.99*	1.77	2.24	1.72*	1.62	1.83	1.07	0.95	1.21
**Sex (reference = female)**															
Male	3.20*	2.87	3.57	5.55*	5.10	6.03	2.45*	2.18	2.75	2.21*	2.08	2.34	2.92*	2.57	3.31
**Year (reference = 2006)**															
2007	1.27*	1.03	1.57	1.26*	1.07	1.47	1.45*	1.13	1.85	1.33*	1.18	1.51	1.02	0.80	1.29
2008	1.69*	1.37	2.07	1.41*	1.21	1.65	1.64*	1.29	2.08	1.35*	1.20	1.53	1.11	0.88	1.40
2009	1.38*	1.13	1.70	1.57*	1.34	1.83	1.60*	1.26	2.04	1.42*	1.25	1.60	0.90	0.71	1.15
2010	1.58*	1.29	1.93	1.80*	1.55	2.10	1.76*	1.39	2.22	1.68*	1.49	1.89	1.32*	1.05	1.67
2011	1.65*	1.34	2.01	1.86*	1.60	2.16	1.92*	1.52	2.43	1.62*	1.44	1.83	1.00	0.79	1.26
2012	1.56*	1.27	1.91	1.68*	1.44	1.96	1.95*	1.54	2.45	1.53*	1.36	1.72	0.85	0.67	1.08
2013	1.42*	1.16	1.74	1.56*	1.34	1.82	1.57*	1.24	1.98	1.31*	1.16	1.48	0.65*	0.50	0.83

*p < 0.05.

MRR, mortality rate ratio; TBI, traumatic brain injury.

The interaction of age group × year was statistically insignificant in all models (all *p*-values for the interaction > 0.05; see Tables 2 and 3) except for the model of the TBI-induced mortality rate for motorcyclists (υ = 42, likelihood ratio = 76.60, *p*-value for the interaction < 0.05).

### External cause of TBI-induced deaths

**Fig 2:**
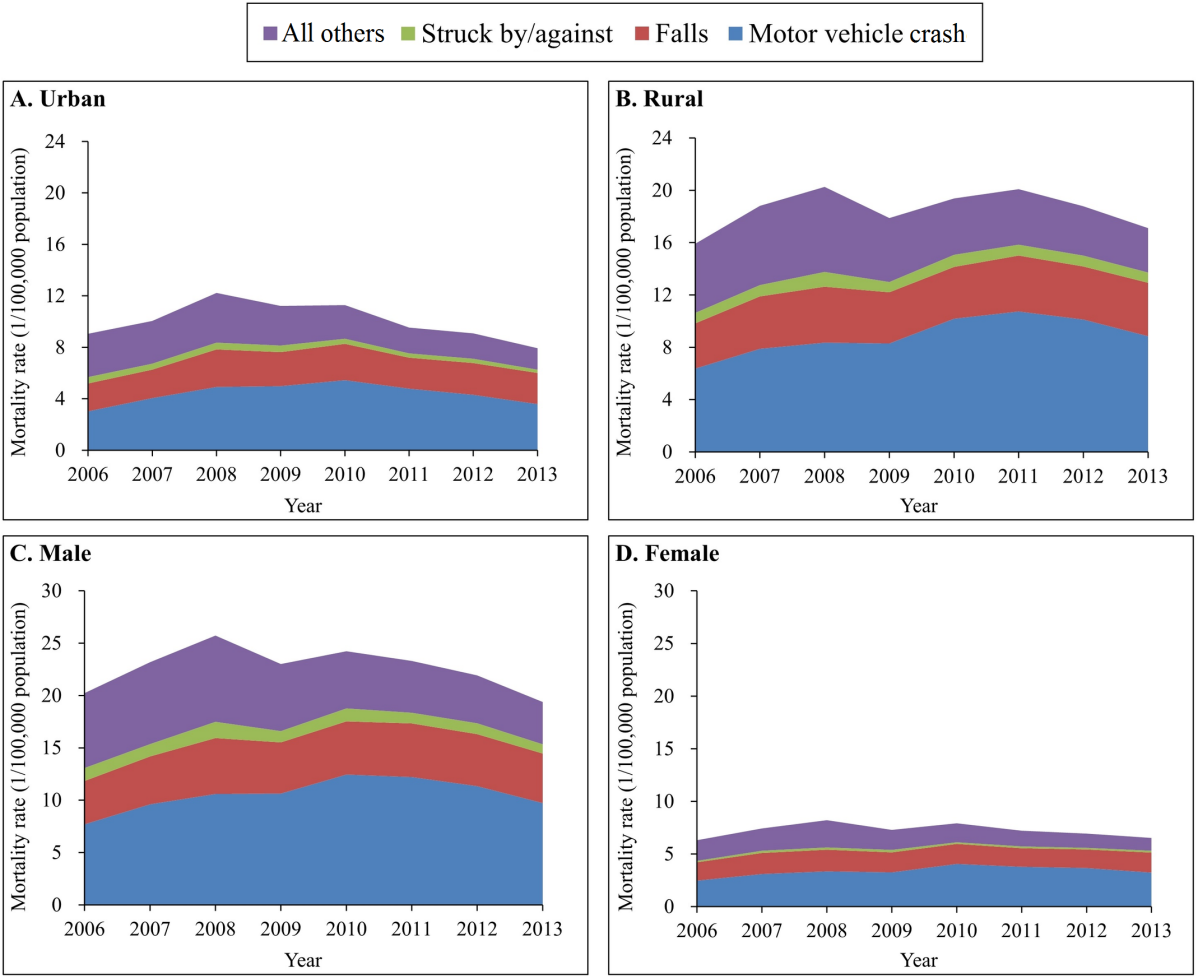
Mortality rates from traumatic brain injury by urban/rural location, sex, and external cause of injury (China, 2006–2013). (A) Urban areas; (B) rural areas; (C) male; (D) female. Mortality rates were age-standardized based on the population of China in 2010.

From 2006–2013, motor vehicle crashes and falls were consistently the 2 leading causes of TBI mortality for both urban and rural residents, and for both males and females (Fig 2). Just as large differences were observed in overall TBI mortality, large urban–rural and male–female gaps also occurred in cause-specific TBI mortality. Strikingly, TBI mortality from motor vehicle crashes generally increased from 2006 to 2010 but began to decrease gradually after 2010 in both urban and rural areas. The reduction since 2010 was greater in urban areas than in rural areas.

**Fig 3:**
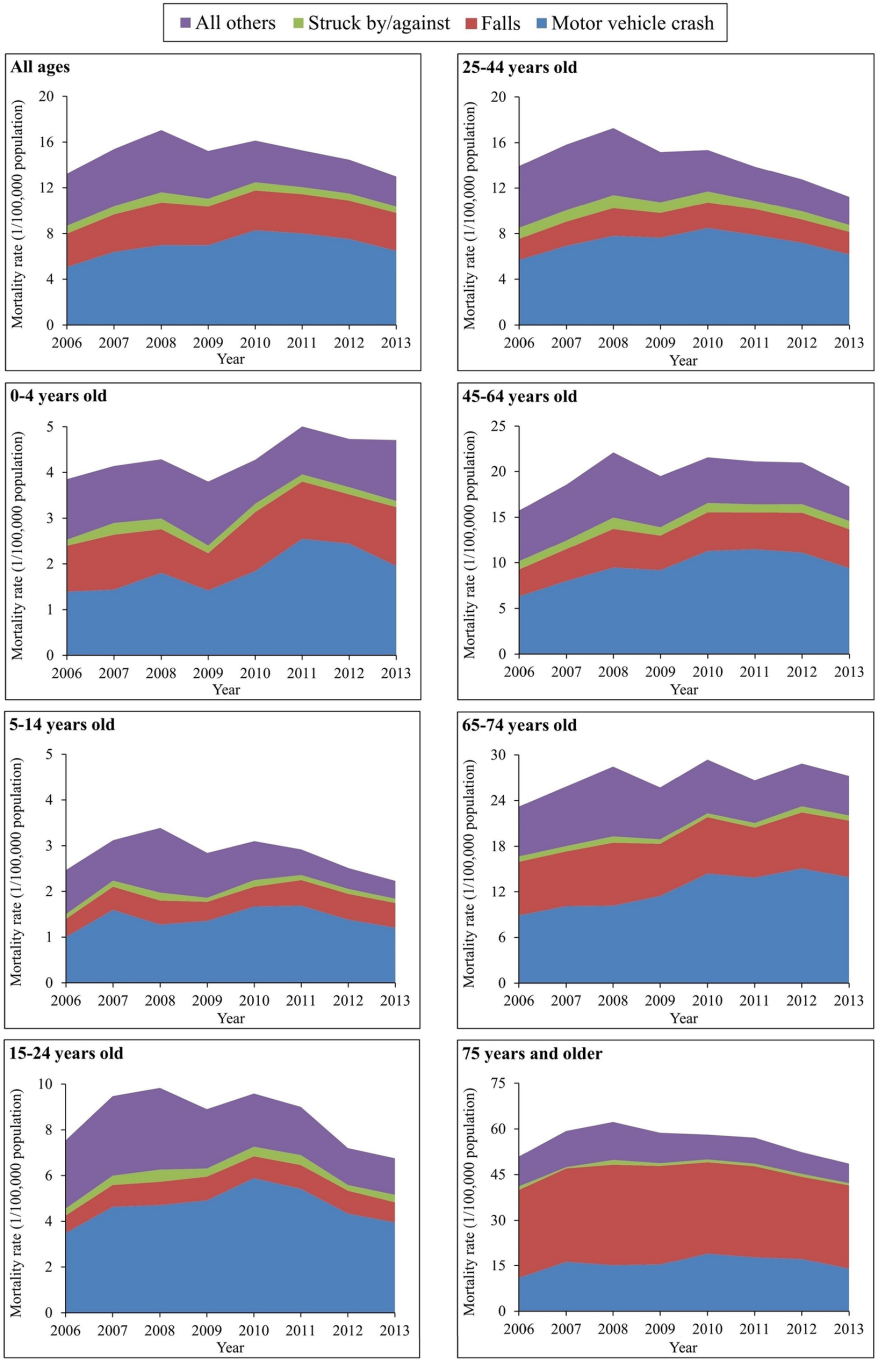
Mortality rates from traumatic brain injury by age group and external cause of injury (China, 2006–2013).

The spectrum of the causes of TBI deaths varied somewhat across age groups. For all 6 age groups less than 75 years, the leading cause of TBI mortality was motor vehicle crashes, but for adults aged 75 years and older, the leading cause was falls (Fig 3). After age 15 years, falls began to contribute more and more to total TBI mortality as age increased.

The external causes of TBI mortality changed greatly between 2006 and 2013 across age groups. All 7 groups showed significant increases in TBI mortality attributed to motor vehicle crashes, with the largest increase occurring in the age group 65–74 years (from 8.89 to 13.95 per 100,000 population) (S5 Table).

**Fig 4:**
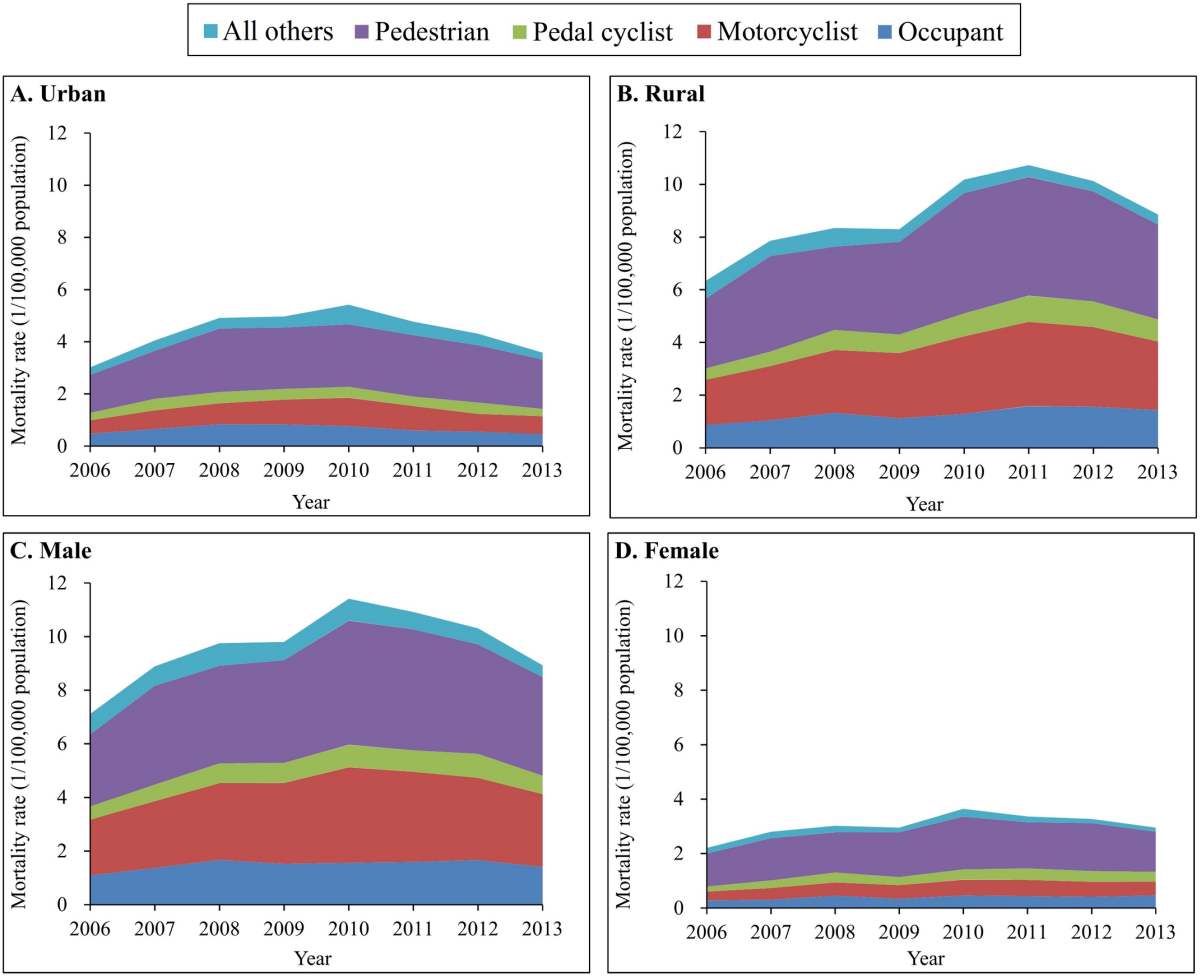
Mortality rates from traumatic brain injury due to motor vehicle crashes by urban/rural location, sex, and road user category (China, 2006–2013). (A) Urban areas; (B) rural areas; (C) male; (D) female. Mortality rates were age-standardized based on the population of China in 2010.

Pedestrians were the most vulnerable road users for TBI deaths attributed to motor vehicle crashes in both urban and rural areas and for both sexes (Fig 4). Notably, the age-adjusted TBI mortality for motorcyclists increased 54% for rural residents (from 1.70 to 2.61 per 100,000 population) and 35% for males (from 2.16 to 2.91 per 100,000 population) between 2006 and 2013. Similarly, TBI mortality for pedal cyclists rose by 91% in rural areas, 41% in males, and 100% in females between 2006 and 2013 (S6 and S7 Tables).

**Fig 5:**
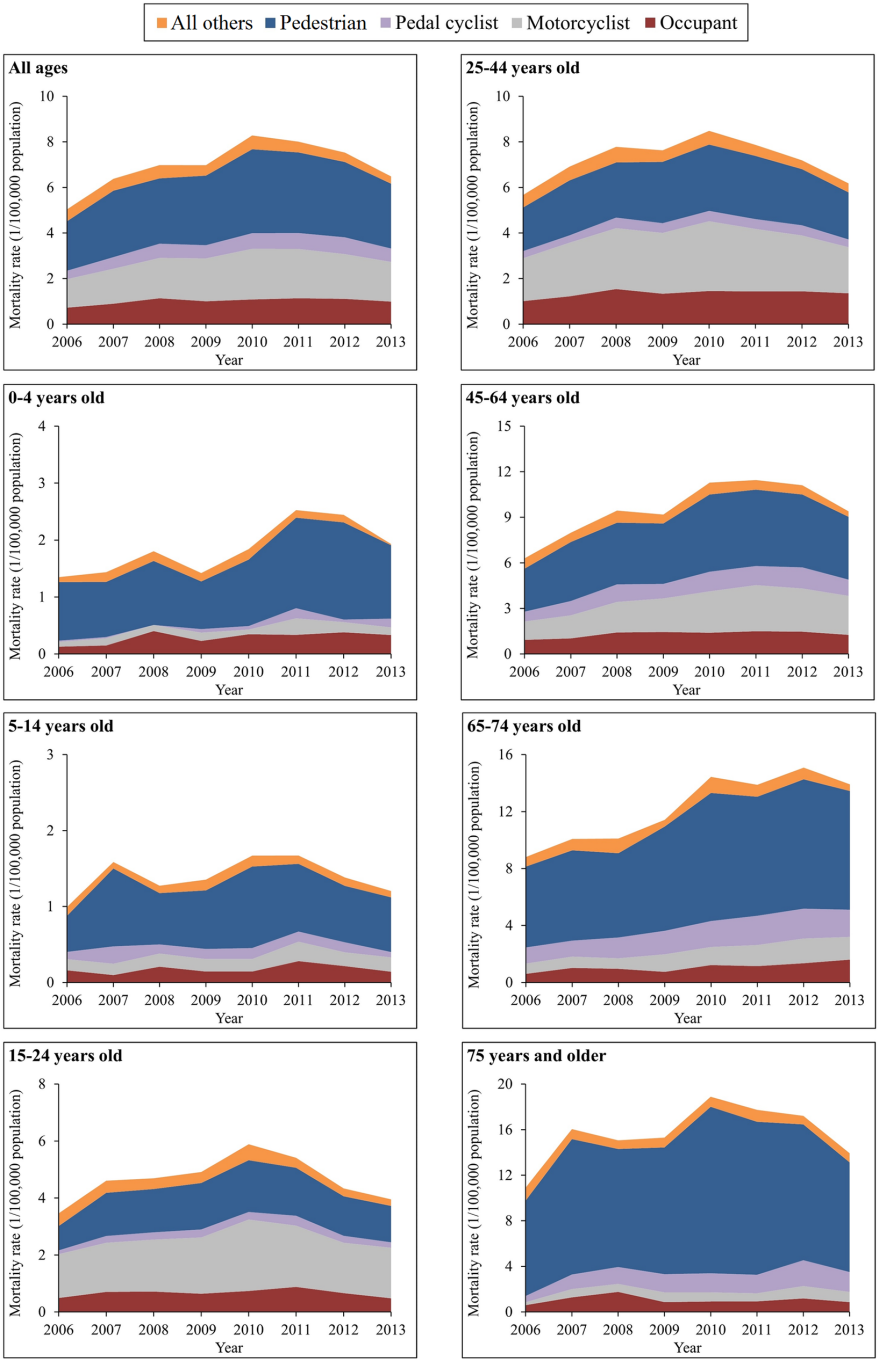
Mortality rates from traumatic brain injury due to motor vehicle crashes by age group and road user category (China, 2006–2013).

The spectrum of road use for TBI mortality attributed to motor vehicle crashes varied across age groups. For children aged 0–4 and 5–14 years and for adults aged 65 years and older, pedestrian deaths constituted over 60% of total TBI deaths from motor vehicle crashes (Fig 5). For the age groups 15–24, 25–44, and 45–64 years, motorcyclist deaths emerged as the first or second leading cause of TBI mortality from motor vehicle crashes, explaining 50% of total TBI deaths due to motor vehicle crashes in 2013; the contribution of motorcyclist deaths to TBI mortality caused by motor vehicle crashes was 59% in the age group 15–24 years (Fig 5).

Across all ages, TBI mortality increased by 38% for vehicle occupants, 39% for motorcyclists, and 31% for pedestrians between 2006 and 2013 (S8 Table). Notable age-specific TBI mortality changes over the study time period included the following: a 166% increase for occupants in the age group 65–74 years, an increase of over 110% for motorcyclists in the age groups ≥ 45 years combined, a 626% rise for pedal cyclists in the age group 0–4 years but a 26% decrease in the age group 5–14 years, and increases for pedestrians in all age groups, especially in the age groups 15–24 years, 45–64 years, and 65–74 years (increased 50%, 46%, and 47%, respectively) (S8 Table).

## Discussion

### Summary of findings

To our knowledge, this is the first published national study to report socio-demographic patterns, trends, and external causes of TBI mortality in a low- or middle-income country like China. Five key findings are summarized as follows: (1) overall TBI mortality remained relatively stable and high (12.99–17.06 per 100,000 population) from 2006 to 2013; (2) there were great variations in TBI rates across locations (urban/rural), sex, and age groups that persisted across the study time period for both overall and cause-specific TBI mortality rates; (3) motor vehicle crashes were the leading cause of TBI mortality for Chinese residents under 75 years, but falls were the leading cause of TBI deaths for adults aged 75 years and older; (4) pedestrians were the most common type of road user in TBI deaths from motor vehicle crashes in children aged 0–14 years and adults aged 65 years and older, but motorcyclists were as vulnerable as pedestrians among residents 15–64 years; and (5) notable TBI mortality increases occurred in persons aged 45–74 years for vulnerable road users (pedestrians, motorcyclists, and pedal cyclists).

### Potential influence of disease surveillance points system updates

As described above, the DSPs have experienced 2 major updates in history. First, there were adjustments made to the surveillance points in 1989 and in 2004–2006. In both cases, the aim was to improve the coverage and representativeness of the surveillance samples for the total population [19–21]. By 2006, the number of surveillance points was raised to 161 points, including 64 urban points and 97 rural points, across 31 provinces [19]. We initiated our study timeline in 2006, after the changes had been implemented, and therefore we assume the changes in surveillance points would not have had a substantial impact on our results.

The second major update to DSPs was the introduction of web-based reporting in 2008 [33]. Since then, all death cases have been reported by the web-based National Death Registration Management System of Chinese CDC. The change in data reporting approach may have caused some problems in practice and may have had an unexpected impact on reported mortality rates. For example, many local employees who were responsible for DSPs data reporting needed time to familiarize themselves with the web-based reporting system (although they had received specific training before the web-based reporting system was introduced nationwide). In addition, the quality control office needed time to develop new methods to assess and guarantee the quality of web-based reporting. Consequently, the introduction of the web-based reporting system in 2008 may have caused unexpected fluctuations in mortality rates in the initial stages after the change. Our results show a notable and consistent fluctuation in overall and location-, sex-, age-, and cause-specific TBI mortality rates between 2007 and 2009. Similar fluctuations are reported in previous publications that used DSPs data from the same period to assess trends in mortality from cardiovascular diseases [22], tuberculosis [23], and road traffic crashes [34,35]. These fluctuations may reflect the effect of the introduction of the web-based reporting system in all DSPs surveillance points in 2008.

### Interpretation of findings

The overall TBI mortality rate in China (12.99 per 100,000 population in 2013 and slightly higher in previous years) approximates that in the US (17.0 per 100,000 population) [6], Europe (11.7 per 100,000 population) [7], and Germany (11.5 per 100,000 population) [36]. Given potential differences across the studies—including aspects of the study methodology, population structure and culture, data acquisition, and definition of TBI [37,38]—differences in TBI mortality observed across countries and regions should be interpreted with caution. However, the data clearly indicate that TBI should be regarded as a significant public health problem in China, as TBI mortality rates remained fairly stable and high from 2006 to 2013.

The slight decrease in TBI mortality since 2010 in China may reflect improvements in pre-hospital aids and hospital treatment, as well as fragmented efforts for injury prevention in China [39–41]. An analysis of pre-hospital aid data from Fangshan District, Beijing, for example, showed that the median time from receiving an emergency call to the arrival of an ambulance decreased significantly between 2009 and 2012 [42]. Based on data from the Chinese Health Statistics Yearbook [43,44], the number of hospital beds per 1,000 population increased from 2.72 beds in 2006 to 4.55 beds in 2013.

Corresponding to results from previous epidemiological studies [3,8] and literature reviews [2,37,45] from HICs, our results showed high TBI mortality risk in Chinese males, rural residents, and older adults. Greater TBI mortality risk in males may primarily reflect more risk-taking behaviors among men than among women, including risks such as distracted driving from cell phone use (68% versus 56%) [46], driving without helmets (62% versus 46%) and seatbelts (27% versus 25%) [47], use of weapons (incidence rate ratio = 2.74) [48], higher alcohol consumption (both in frequency and quantity) [49], and driving under the influence of alcohol (odds ratio: 2.78) [50]. Our finding of increased risk among rural residents also replicates results from previous studies [51–54]. Higher risk among rural residents may be attributed to more law violations and high-risk behaviors of rural residents, plus underdeveloped injury prevention and medical care in rural areas. Finally, our study reproduces findings from HICs [28,36,55] that older adults have higher TBI mortality risk. Such a finding is likely the result of the natural aging process. As adults age, they are at increased risk of having multiple chronic diseases such as hypertension, hyperlipidemia, and glaucoma, which result in balance impairments, vision disorders, and/or cognitive impairment and raise the risk of TBI injury. According to the Global Burden of Disease Study 2013, the number of individuals with several morbidities rapidly increases with age: in elderly adults aged 80 years and older, 10.3% had 1 to 4 sequelae, 64.6% had 5 to 9 sequelae, and 25.1% had 10 or more sequelae in 2013 [56].

One finding differs substantially from reports in other countries. In China, motor vehicle crashes were the most common cause of TBI mortality for all age groups under 75 years, in contrast to cause patterns in the United States, where firearm-related wounds are the leading cause of TBI mortality in the age groups 20–24 and ≥75 years [28], and in contrast to many European countries, including Austria, Finland, Germany, Switzerland, and the United Kingdom, where about 50% of TBI mortalities are caused by falls [7]. Our finding represents the heavy burden caused by road traffic crashes to Chinese residents, and the urgent need for motor vehicle safety efforts. These results concord with those from published domestic epidemiological studies for overall road traffic mortality in China [34,35,57,58].

Within the motor vehicle crash category, our detailed analysis by road user category reveals important findings. Chinese children aged 0–4 and 5–14 years, as well as adults aged 65 years and older, were most vulnerable to TBI death as pedestrians. In the middle years of life (ages 15–64 years), motorcyclists emerged as the road users with the highest rate of TBI death. These results correspond with all-mortality data among road users in other LMICs but differ from data from HICs [45]. They likely reflect the patterns of road use in China across different age groups, and the distinctions in road use and motorcycle operation between LMICs and HICs. In addition, we also observed distinct urban–rural differences in TBI mortality patterns, which were mainly driven by urban–rural differences in mortality from motor vehicle crashes. These results agree with the analysis for total road traffic mortality [54].

### Implications

Our findings have 2 major implications. First, the detailed analysis quantifies the severity of TBI as a public health problem in China. It describes high-risk populations and the major causes of TBI mortality, providing rigorous empirical evidence for decision-makers to tackle this problem and offering valuable data to estimate global TBI burdens. The results also highlight the urgency of improving injury prevention, pre-hospital aid, hospital treatment, and recovery after experiencing a TBI.

The primary and urgent strategic goal for enhancing injury prevention is to assign a single government agency to coordinate injury prevention efforts that presently span multiple government departments and partners [39–41]. From the perspective of immediate action, some prevention measures that have been demonstrated effective in HICs should be introduced and tailored culturally to ensure their acceptability, feasibility, and efficacy. Examples include helmet use for bicyclists and motorcyclists, child restraint (car seat and booster seat) use for child passengers, seatbelt use for all vehicle passengers, use of shock-absorbing materials in the surface of children’s playgrounds, and periodic review of vision, medication, and home environment assessments and modifications [59–62] to reduce fall-induced injury, especially among older adults. Additionally, government policies to improve the safety of hospitals and nursing facilities and to enhance the pension policy and system for the older people, especially elderly individuals in rural areas, have potential to play a meaningful role in reducing the morbidity and mortality of TBI caused by falls [63]. Unfortunately, many proven interventions to prevent major causes of unintentional child injuries are underrepresented in Chinese laws and regulations, and the associated implementation responsibilities are poorly defined [64]. Basic prerequisites to implement proven prevention interventions should be implemented by the government of China.

In addition, effective interventions to improve pre-hospital aid, hospital treatment, and rehabilitation services for TBI patients are needed [65–67]. These may include developing a pre-hospital trauma care system with reference to successful programs in HICs, quality improvement programs, speeding up rescue efforts to ensure individuals receive life-sustaining care within a few minutes of injury, and teaching individuals how to react at the scene of an injury. These programs can prevent TBI-related death and disability and should be introduced and disseminated nationwide. Of course, necessary tailoring and modifications may be incorporated to respect China’s healthcare system and culture.

Second, our results provide valuable data to guide etiological studies exploring causal and modifiable factors leading to high TBI mortality rates particularly in Chinese populations, including vulnerable and high-risk populations (males, rural residents, and older adults). Our study also offers data concerning significant rises in TBI mortality among specific groups, such as vulnerable road users in the age group ≥75 years. In addition, effective, easy-to-implement interventions should be applied in practice to protect high-risk populations. Such interventions could include encouraging vulnerable pedestrians to wear light-colored clothing and reflective materials, construction of sidewalks and raised crossings, reducing legal speed limits, and construction of refuge islands and raised platforms at bus stops [68,69].

### Study limitations

This study has several limitations. First, the DSPs dataset does not cover confounding factors that influence TBI mortality and may influence TBI mortality trends. Such factors include individual differences and behaviors, environmental factors, and pre-hospital and hospital care. Second, TBI morbidity data were not available. Morbidity data may reveal different trends, patterns, and cause spectrums compared with mortality data and are needed within LMICs. Third, TBI deaths may be seriously underestimated in the DSPs mortality data because of many missing S- or T-codes in injury-related deaths. A TBI death in the DSPs system is defined only with the coexistence of an external cause of injury code (E-code) and a nature of injury code (N-code, namely S- or T-code). Supplemental analysis shows that up to 46% of injury deaths of 2006–2013 did not have an S- or -T-code on the death certificate, a proportion that is far higher than that in the US (0.6%) [70]. This strongly suggests that TBI deaths may be underestimated in our analysis. Further, we found that the proportion of missing S- or T-codes increased slightly between 2009 and 2013, which may have led to an apparent decrease of TBI mortality rates and influenced TBI mortality trends to a small extent. Finally, our results may be affected by the completeness of reporting, including the accuracy of coding of cause of death in the DSPs system. The final data were rigorously checked and corrected through various methods including routine national survey sampling every 3 years at all surveillance points [71,72], but minor flaws may remain.

### Conclusion

In conclusion, TBI mortality has remained high and stable for many years in China, constituting a significant threat to the health of the Chinese population. Males and rural residents are at particularly high risk of dying from TBI compared to females and urban residents. TBI mortality risk rises dramatically as individuals age. Future research should explore reasons for the particularly high risk of TBI mortality among particular populations, as well as for recent increases in certain subgroups. In particular, evidence-based prevention, response, and treatment interventions for TBI, as recommended by the World Health Organization and other agencies, should be translated to Chinese culture and implemented nationwide. Examples of such programs include efforts to reduce injury risk from motor vehicle crashes (helmet use, car seat and booster seat use, lower speed limits, improved drunk driving laws, and alcohol ignition interlock use) and falls (exercise, home modification, and multifaceted interventions).

## Supporting information

S1 Protocol Research plan written before analyzing the data. (DOCX)

S1 RECORD Checklist The RECORD statement—checklist of items, extended from the STROBE statement, that should be reported in observational studies using routinely collected health data. (DOCX)

S1 Table Sample characteristics of national Disease Surveillance Points system of China, 2006–2013. (DOC)

S2 Table Age-standardized mortality rates from traumatic brain injury per 100,000 population (standard error) by cause and location in China, 2006–2013. (DOCX)

S3 Table Age-standardized mortality rates from traumatic brain injury per 100,000 population (standard error) by cause and sex in China, 2006–2013. (DOCX)

S4 Table Mortality rates from traumatic brain injury per 100,000 population (standard error) by 5-year age group in China, 2006–2013. (DOCX)

S5 Table Mortality rates from traumatic brain injury per 100,000 population (standard error) by cause and age group in China, 2006–2013. (DOCX)

S6 Table Age-standardized mortality rates from traumatic brain injury due to motor vehicle crashes per 100,000 population (standard error) by road user category and location in China, 2006–2013. (DOCX)

S7 Table Age-standardized mortality rates from traumatic brain injury due to motor vehicle crashes per 100,000 population (standard error) by road user category and sex in China, 2006–2013. (DOCX)

S8 Table Mortality rates from traumatic brain injury due to motor vehicle crashes per 100,000 population (standard error) by road user category and age group in China, 2006–2013. (DOCX)

## Abbreviations

CDC: Center[s] for Disease Control and Prevention
DSPs: Disease Surveillance Points
HICs: high-income countries
ICD-10: International Classification of Diseases–10th Revision
LMICs: low- and middle-income countries
MRR: mortality rate ratio
TBI: traumatic brain injury
