# Supplementary material for: Trends in traumatic brain injury mortality in China, 2006–2013: A population-based longitudinal study
Source: PLoS Med. 2017 Jul 11;14(7):e1002332. doi: 10.1371/journal.pmed.1002332 (PMC5507407; doi:10.1371/journal.pmed.1002332)
Supplement: S1 Protocol — (DOCX) [file pmed.1002332.s001.docx]

**Planning written before analyzing the data**

**Title:** Trends in traumatic brain injury mortality in China, 2006-2013

**1. Background**

**1.1 Rationale of conducting this analysis**

1.1.1 Traumatic brain injury (TBI) is an important public health problem worldwide. The incidence is increasing worldwide, particularly in middle-income and low-income countries [1]. It has been estimated that TBI affect over 10 million people annually leading to either mortality or hospitalization [2].

1.1.2 In the past, the studies of traumatic brain injury were mainly based on the hospital data.

1.1.3 This study will describe the number, rate and proportion of traumatic brain injury related death by age, sex, location and year, based on the data form DSP, 2006-2013, China. Also assessed was the change over time of specific external causes of TBI-related death.

**1.2 Research questions**

1.2.1 Is there any difference in number or rate for traumatic brain injury related death between different location, sex, and age groups?

1.2.2 Did overall or cause-specific subgroup injury mortality rates change during 2006-2013? How did they change?

1.2.3 Are there any differences in the proportions of external cause in different age groups and what are the most important external causes of traumatic brain injury for the seven age groups?

**2. Method**

**2.1 Design and data sources**

We will conduct a population-based longitudinal analysis to examine trends in TBI mortality and differences based on location (urban/rural), sex, age group, and external cause. Data will be obtained from the National Diseases Surveillance Points System (DSP), 2006-2013.

The DSPs is a national cause-of-death data system in China and was established by the Chinese central government in 1978 [3]. Two substantial adjustments were made to DSP in 1989 and in 2004-2006 to enhance its national representativeness [4, 5], and in 2013, the number of surveillance points was increased to 605 points [6], but we will use data from the 161 points available beginning in 2006 for this analysis to avoid potential impact of the change of DSP.

All causes of death in DSPs are determined according to a standard protocol by trained coders located in hospitals or local CDC offices. Since 2004, deaths are coded using the ICD-10. Starting in 2008, a web-based reporting system is used in the DSPs system [7, 8]. Quality control methods [3, 9], including internal procedural check system, statistical measures (e.g., the standard United Nations Age Sex Accuracy Index), fixed national sample survey, are conducted to reduce under-reporting and ensure the reliable, valid and nationally representative of DSP.

**2.2 Case definition**

Cases of TBI is identified and selected from parts I and II of the death certificates in which one or more diagnostic codes representing TBI are included in the sequence of conditions contributing to death. TBI-induced deaths are identified according to the ICD-10 codes recommended by U.S. CDC [10, 11], which cover the following diagnosis codes: S01.0-S01.9, S02.0, S02.1, S02.3, S02.7-S02.9, S04.0, S06.0-S06.9, S07.0, S07.1, S07.8, S07.9, S09.7−S09.9, T01.0, T02.0, T04.0, T06.0, T90.1, T90.2, T90.4, T90.5, T90.8 and T90.9. And the following ICD-10 diagnosis codes are used to identify TBI deaths [12]: **(Table 1)**

**Table 1: ICD-10 codes for external cause of traumatic brain injury**

| **Description** | **ICD-10** |
| --- | --- |
| **Motor vehicle crash** |  |
| Occupant | V30−V79 (.4−.9), V81.1, V82.1, V83−V86 (.0−.3) |
| Motorcyclist | V20−V28 (.3−.9), V29 (.4−.9) |
| Pedal cyclist | V12−V14 (.3−.9), V19 (.4−.6) |
| Pedestrian | V02−V04 (.1, .9), V09.2 |
| Other unintentional | V80 (.3−.5), V87 (.0−.8), V89.2 |
| Homicide | Y03 |
| Suicide | X82 |
| Undetermined | Y32 |
| **Falls** |  |
| Unintentional | W00−W19 |
| Homicide | Y01 |
| Suicide | X80 |
| Undetermined | Y30 |
| **Struck by and against** |  |
| Unintentional | W20−W22, W50−W52 |
| Homicide | Y00, Y04 |
| Suicide | X79 |
| Undetermined | Y29, Y35.3 |
| **Other** | All other codes |

**2.3 Study variables**

Data are stratified by year, age, sex, location and external cause.

2.3.1 Year: 2006-2013;

2.3.2 Location: (1) urban, (2) rural

2.3.3 Sex: (1) male, (2) female

2.3.4 Age group:

According to the previous publications [13, 14], we divide age into seven groups, including (1)0-4 years, (2)5-14 years, (3)15-24 years, (4)25-44 years, (5)45-64 years, (6)65-74 years, (7) 75 years and older. Detailed analysis of five-year age group will be included as an appendix (note: Detailed analysis of five-year age group was added later according to the reviewers’ comments).

2.3.5 External cause:

(1) Motor vehicle traffic (including occupant, motorcyclist, pedal cyclist, pedestrian, other unintentional, homicide suicide and undetermined),

(2) Fall (including unintentional, homicide, suicide and undetermined),

(3) Struck by and against (including unintentional, homicide, suicide and undetermined);

And (4) all other causes.

**2.4 Statistical analysis**

2.4.1 Overall and subgroup-specific (urban/rural, male/female, major injury causes and road users) age-standardized mortality rates (95% CI). Census population in 2010 will be used as reference population to calculate age-standardized mortality.

2.4.2 Age-specific mortality rates (95% CI) for seven age groups;

2.4.3 Pearson Chi-square test will be used to determine whether there is a difference in the mortality rates between subgroups. When the assumption for chi-square test is violated, Fisher’s exact test will be used.

2.4.4 The Cochran-Armitage trend test will be used to determine the significance of mortality change over time.

2.4.5 Negative binomial models [15] will be used to examine the associations of deaths with socio-demographic factors. Mortality rate ratios (MRR) and corresponding 95% CIs will quantify the extent of associations.

2.4.7 Likelihood ratio test will be used to test the significance of the interaction of age group*year (note: The analysis of interaction was added later according to the reviewers’ comments).

2.4.6 *p* values < 0.05 will be considered statistically significant.

**3. Expected Results**

**Table 1:** Mortality rates of traumatic brain injury by location, sex, age group and external cause in China, 2006-2013

Note: The overall and annual TBI induced mortality rates will been listed in this table by location, sex, age groups and external causes between 2006 and 2013. In addition, we will conduct Cochran-Armitage trend tests to examine the significance of change over time during the interval.

**Table 2:** Multivariate negative binomial regression

**Figure 1:** Mortality rates from traumatic brain injury by location (urban/rural), sex and age group in China, 2006-2013

Note: Line graph.

**Figure 2:** Mortality rates from traumatic brain injury by age group and external cause (China, 2006-2013)

Note: Cumulative area chart

**Figure 3:** Mortality rates from traumatic brain injury due to motor vehicle crash by age group and road users (China, 2006-2013)

Note: Cumulative area chart

**Figure 4:** Mortality rates from traumatic brain injury by location, sex and external cause (China, 2006-2013)

Note: Cumulative area chart

**Figure 5**: Mortality rates from traumatic brain injury due to motor vehicle crash by location, sex and road users (China, 2006-2013)

Note: Cumulative area chart

**Reference**

1. Hyder AA, Wunderlich CA, Puvanachandra P, Gururaj G, Kobusingye OC. The impact of traumatic brain injuries: a global perspective. Neurorehabilitation. 2007; 22: 341-53.

2. Stocchetti N. Traumatic brain injury: problems and opportunities. Lancet Neurol, 2014, 13(1):14-6.

3. Yang G, Hu J, Rao KQ, Ma J, Rao C, Lopez AD. Mortality registration and surveillance in China: History, current situation and challenges. Popul Health Metr, 2005, 3:3.

4. Zhou M, Jiang Y, Huang Z, Wu F. Adjustment and representativeness evaluation of national disease surveillance points system. Disease Surveillance. 2010; 25: 239-44.

5. Mooney P. Counting the dead in China. Bull World Health Organ. 2006; 84: 168-9.

6. National Center for Chronic and Non-communicable Disease Control and Prevention, Chinese Center for Disease Control and Prevention. Data sets of death cause surveillance in disease surveillance points system, 2013. Beijing: Popular Science Press; 2015.

7. Liu MB, Wang W, Zhou MG. Trend analysis on the mortality of cardiovascular diseases from 2004 to 2010 in China. Zhonghua Liu Xing Bing Xue Za Zhi. 2013; 34: 985-8.

8. Zhang H, Huang F, Chen W, Du X, Zhou MG, Hu J, et al. Estimates of Tuberculosis Mortality Rates in China Using the Disease Surveillance Point System, 2004-2010. Biomed Environ Sci. 2012; 25: 483-8.

9. Wang L, Wang LJ, Cai Y, Ma LM, Zhou MG. Analysis of under-reporting of mortality surveillance from 2006 to 2008 in China. Zhonghua Yu Fang Yi Xue Za Zhi. 2011; 45: 1061-4.

10. Faul M, Xu L, Wald MM, Coronado VG. Traumatic Brain Injury in the United States: Emergency Department Visits, Hospitalizations and Deaths. Atlanta (GA): Centers for Disease Control and Prevention, National Center for Injury Prevention and Control; 2010.

11. Thurman DJ, Sniezek JE, Johnson D, Greenspan A, Smith SM. Guidelines for surveillance of central nervous system injury. Atlanta (GA): US Department of Health and Human Services, Public Health Service, CDC; 1995.

12. Coronado VG, Xu L, Basavaraju SV, McGuire LC, Wald MM, Faul MD, et al. Surveillance for traumatic brain injury-related deaths--United States, 1997-2007. MMWR Surveill Summ. 2011; 60: 1-32.

13. Feigin VL, Theadom A, Barker-Collo S, Starkey NJ, McPherson K, Kahan M, et al. Incidence of traumatic brain injury in New Zealand: a population-based study. Lancet Neurol. 2013; 12: 53-64.

14. Faul M, Xu L, Wald MM, Coronado VG. Traumatic Brain Injury in the United States: Emergency Department Visits, Hospitalizations and Deaths. Atlanta (GA): Centers for Disease Control and Prevention, National Center for Injury Prevention and Control; 2010.

15. Statistical Consulting Group. Annotated Stata Output: Negative Binomial Regression. 2012. Available from: http://www.ats.ucla.edu/stat/stata/output/stata_nbreg_output.htm. Cited 17 September 2016.
