# Supplementary material for: Trends in traumatic brain injury mortality in China, 2006–2013: A population-based longitudinal study
Source: PLoS Med. 2017 Jul 11;14(7):e1002332. doi: 10.1371/journal.pmed.1002332 (PMC5507407; doi:10.1371/journal.pmed.1002332)
Supplement: S1 Table — (DOC) [file pmed.1002332.s003.doc]

**S****upplementary Table 1. Sample characteristics of national Disease Surveillance Points of China, 2006-2013**

| **Variable** | **2006** | |  | **2007** | |  | **2008** | |  | **2009** | |  | **2010** | |  | **2011** | |  | **2012** | |  | **2013** | |
| --- | --- | --- | --- | --- | --- | --- | --- | --- | --- | --- | --- | --- | --- | --- | --- | --- | --- | --- | --- | --- | --- | --- | --- |
|  | **Number** | **%** |  | **Number** | **%** |  | **Number** | **%** |  | **Number** | **%** |  | **Number** | **%** |  | **Number** | **%** |  | **Number** | **%** |  | **Number** | **%** |
| **Total** | 76173847 | 100 |  | 76647996 | 100 |  | 77417436 | 100 |  | 77994298 | 100 |  | 78287479 | 100 |  | 81533690 | 100 |  | 81896879 | 100 |  | 82753345 | 100 |
| **Location** |  |  |  |  |  |  |  |  |  |  |  |  |  |  |  |  |  |  |  |  |  |  |  |
| Urban | 28509454 | 37.4 |  | 28688427 | 37.4 |  | 29174329 | 37.7 |  | 29385882 | 37.7 |  | 29334727 | 37.5 |  | 34985398 | 42.9 |  | 35425299 | 43.3 |  | 36002020 | 43.5 |
| Rural | 47664393 | 62.6 |  | 47959569 | 62.6 |  | 48243107 | 62.3 |  | 48608416 | 62.3 |  | 48952752 | 62.5 |  | 46548292 | 57.1 |  | 46471580 | 56.7 |  | 46751325 | 56.5 |
| **Sex** |  |  |  |  |  |  |  |  |  |  |  |  |  |  |  |  |  |  |  |  |  |  |  |
| Male | 38329594 | 50.3 |  | 39153205 | 51.1 |  | 39524734 | 51.1 |  | 39798517 | 51.0 |  | 39932567 | 51.0 |  | 41513993 | 50.9 |  | 41749030 | 51.0 |  | 42180929 | 51.0 |
| Female | 37844253 | 49.7 |  | 37494791 | 48.9 |  | 37892702 | 49.0 |  | 38195781 | 49.0 |  | 38354912 | 49.0 |  | 40019697 | 49.1 |  | 40147849 | 49.0 |  | 40572416 | 49.0 |
| **Age group** |  |  |  |  |  |  |  |  |  |  |  |  |  |  |  |  |  |  |  |  |  |  |  |
| 0-4 years | 4669101 | 6.1 |  | 4661656 | 6.1 |  | 4711606 | 6.1 |  | 4789324 | 6.1 |  | 4884253 | 6.2 |  | 4474223 | 5.5 |  | 4460001 | 5.5 |  | 4502551 | 5.4 |
| 5-14 years | 9445417 | 12.4 |  | 9268518 | 12.1 |  | 9174757 | 11.9 |  | 9079324 | 11.6 |  | 9038306 | 11.6 |  | 8191257 | 10.1 |  | 8326046 | 10.2 |  | 8473897 | 10.2 |
| 15-24 years | 13400859 | 17.6 |  | 13457212 | 17.6 |  | 13256799 | 17.1 |  | 13144966 | 16.9 |  | 12877198 | 16.5 |  | 13414162 | 16.5 |  | 13345930 | 16.3 |  | 13056742 | 15.8 |
| 25-44 years | 25489806 | 33.5 |  | 25874180 | 33.8 |  | 26016689 | 33.6 |  | 25500513 | 32.7 |  | 25166980 | 32.2 |  | 26969349 | 33.1 |  | 26680581 | 32.6 |  | 25975414 | 31.4 |
| 45-64 years | 17030063 | 22.4 |  | 17123569 | 22.3 |  | 17857926 | 23.1 |  | 18978716 | 24.3 |  | 19717362 | 25.2 |  | 21051596 | 25.8 |  | 21476676 | 26.2 |  | 22891787 | 27.7 |
| 65-74 years | 4141360 | 5.4 |  | 4225850 | 5.5 |  | 4294891 | 5.6 |  | 4298913 | 5.5 |  | 4337761 | 5.5 |  | 4862398 | 6.0 |  | 4660918 | 5.7 |  | 4737769 | 5.7 |
| ≥ 75 years | 1997241 | 2.6 |  | 2037011 | 2.7 |  | 2104768 | 2.7 |  | 2202542 | 2.8 |  | 2265619 | 2.9 |  | 2570705 | 3.2 |  | 2946727 | 3.6 |  | 3115185 | 3.8 |

Note: The sample of DSPs was representative to the whole country for all years.
