# Supplementary material for: Trends in traumatic brain injury mortality in China, 2006–2013: A population-based longitudinal study
Source: PLoS Med. 2017 Jul 11;14(7):e1002332. doi: 10.1371/journal.pmed.1002332 (PMC5507407; doi:10.1371/journal.pmed.1002332)
Supplement: S2 Table — (DOCX) [file pmed.1002332.s004.docx]

**Supplementary Table 2. Age-standardized mortality rates from traumatic brain injury per 100,000 population (standard error) by cause and location in China, 2006-2013**

| **Cause** | **Location** | **2006** | **2007** | **2008** | **2009** | **2010** | **2011** | **2012** | **2013** | **% change in rate** |
| --- | --- | --- | --- | --- | --- | --- | --- | --- | --- | --- |
| **All causes** | Urban | 9.06 (0.18) | 10.05 (0.19) | 12.23 (0.20) | 11.22 (0.20) | 11.27 (0.20) | 9.54 (0.17) | 9.08 (0.16) | 7.94 (0.15) | -12^**^ |
|  | Rural | 15.91 (0.18) | 18.82 (0.20) | 20.26 (0.20) | 17.89 (0.19) | 19.37 (0.20) | 20.10 (0.21) | 18.78 (0.20) | 17.11 (0.19) | 8^**^ |
|  | Ratio | 1.8 | 1.9 | 1.7 | 1.6 | 1.7 | 2.1 | 2.1 | 2.2 |  |
| **Motor vehicle crash** | Urban | 3.03 (0.10) | 4.06 (0.12) | 4.92 (0.13) | 4.98 (0.13) | 5.44 (0.14) | 4.78 (0.12) | 4.31 (0.11) | 3.58 (0.10) | 18^**^ |
|  | Rural | 6.38 (0.12) | 7.88 (0.13) | 8.35 (0.13) | 8.30 (0.13) | 10.18 (0.14) | 10.73 (0.15) | 10.13 (0.15) | 8.85 (0.14) | 39^**^ |
|  | Ratio | 2.1 | 1.9 | 1.7 | 1.7 | 1.9 | 2.2 | 2.4 | 2.5 |  |
| **Falls** | Urban | 2.16 (0.09) | 2.21 (0.09) | 2.92 (0.10) | 2.65 (0.09) | 2.82 (0.10) | 2.41 (0.08) | 2.47 (0.08) | 2.42 (0.08) | 12^*^ |
|  | Rural | 3.45 (0.09) | 4.01 (0.09) | 4.27 (0.09) | 3.91 (0.09) | 3.97 (0.09) | 4.27 (0.10) | 4.05 (0.09) | 4.07 (0.09) | 18^**^ |
|  | Ratio | 1.6 | 1.8 | 1.5 | 1.5 | 1.4 | 1.8 | 1.6 | 1.7 |  |
| **Struck by/against** | Urban | 0.50 (0.04) | 0.46 (0.04) | 0.53 (0.04) | 0.52 (0.04) | 0.41 (0.04) | 0.33 (0.03) | 0.34 (0.03) | 0.25 (0.03) | -50^**^ |
|  | Rural | 0.81 (0.04) | 0.87 (0.04) | 1.13 (0.05) | 0.78 (0.04) | 0.93 (0.04) | 0.85 (0.04) | 0.83 (0.04) | 0.79 (0.04) | -2 |
|  | Ratio | 1.6 | 1.9 | 2.1 | 1.5 | 2.3 | 2.6 | 2.4 | 3.2 |  |
| **All others** | Urban | 3.37 (0.11) | 3.32 (0.11) | 3.86 (0.12) | 3.07 (0.10) | 2.61 (0.09) | 2.02 (0.08) | 1.96 (0.07) | 1.68 (0.07) | -50^**^ |
|  | Rural | 5.27 (0.11) | 6.06 (0.11) | 6.50 (0.12) | 4.90 (0.10) | 4.30 (0.09) | 4.24 (0.1) | 3.77 (0.09) | 3.39 (0.09) | -36^**^ |
|  | Ratio | 1.6 | 1.8 | 1.7 | 1.6 | 1.6 | 2.1 | 1.9 | 2.0 |  |

Notes:

1: Percent change in rate was calculated as “(mortality in 2013- mortality in 2006)/(mortality in 2006)×100”.

2: ^*^: *p*<0.05; ^**^: *p*<0.01.
