# Supplementary material for: Trends in traumatic brain injury mortality in China, 2006–2013: A population-based longitudinal study
Source: PLoS Med. 2017 Jul 11;14(7):e1002332. doi: 10.1371/journal.pmed.1002332 (PMC5507407; doi:10.1371/journal.pmed.1002332)
Supplement: S3 Table — (DOCX) [file pmed.1002332.s005.docx]

**Supplementary Table 3. Age-standardized mortality rates from traumatic brain injury per 100,000 population (standard error) by cause and sex in China, 2006-2013**

| **Cause** | **Sex** | **2006** | **2007** | **2008** | **2009** | **2010** | **2011** | **2012** | **2013** | **% change in rate** |
| --- | --- | --- | --- | --- | --- | --- | --- | --- | --- | --- |
| **All causes** | Male | 20.23 (0.23) | 23.19 (0.24) | 25.74 (0.26) | 23.02 (0.24) | 24.23 (0.25) | 23.31 (0.24) | 21.92 (0.23) | 19.38 (0.23) | -4^**^ |
|  | Female | 6.30 (0.13) | 7.43 (0.14) | 8.22 (0.15) | 7.29 (0.14) | 7.91 (0.14) | 7.20 (0.13) | 6.94 (0.13) | 6.53 (0.13) | 4 |
|  | Ratio | 3.2 | 3.1 | 3.1 | 3.2 | 3.1 | 3.2 | 3.2 | 3.0 |  |
| **Motor vehicle crash** | Male | 7.69 (0.14) | 9.62 (0.16) | 10.6 (0.16) | 10.63 (0.16) | 12.44 (0.18) | 12.21 (0.17) | 11.35 (0.16) | 9.72 (0.16) | 26^**^ |
|  | Female | 2.47 (0.08) | 3.10 (0.09) | 3.34 (0.09) | 3.26 (0.09) | 4.06 (0.10) | 3.78 (0.10) | 3.67 (0.10) | 3.22 (0.10) | 30^**^ |
|  | Ratio | 3.4 | 3.1 | 3.2 | 3.3 | 3.1 | 3.2 | 3.1 | 3.0 |  |
| **Falls** | Male | 4.15 (0.10) | 4.57 (0.11) | 5.34 (0.12) | 4.88 (0.11) | 5.08 (0.11) | 5.13 (0.11) | 4.96 (0.11) | 4.73 (0.11) | 14^**^ |
|  | Female | 1.74 (0.07) | 2.00 (0.07) | 2.06 (0.07) | 1.89 (0.07) | 1.88 (0.07) | 1.76 (0.07) | 1.76 (0.07) | 1.91 (0.07) | 10 |
|  | Ratio | 2.4 | 2.3 | 2.6 | 2.6 | 2.7 | 2.9 | 2.8 | 2.5 |  |
| **Struck by/against** | Male | 1.24 (0.06) | 1.19 (0.06) | 1.55 (0.06) | 1.10 (0.05) | 1.25 (0.06) | 1.02 (0.05) | 1.04 (0.05) | 0.91 (0.05) | -27^**^ |
|  | Female | 0.14 (0.02) | 0.21 (0.02) | 0.22 (0.02) | 0.24 (0.03) | 0.17 (0.02) | 0.18 (0.02) | 0.16 (0.02) | 0.18 (0.02) | 29 |
|  | Ratio | 8.9 | 5.7 | 7.0 | 4.6 | 7.4 | 5.7 | 6.5 | 5.1 |  |
| **All others** | Male | 7.16 (0.14) | 7.81 (0.14) | 8.25 (0.14) | 6.41 (0.13) | 5.45 (0.12) | 4.96 (0.11) | 4.57 (0.10) | 4.03 (0.10) | -44^**^ |
|  | Female | 1.95 (0.07) | 2.12 (0.08) | 2.59 (0.08) | 1.9 (0.07) | 1.79 (0.07) | 1.48 (0.06) | 1.35 (0.06) | 1.21 (0.06) | -38^**^ |
|  | Ratio | 3.7 | 3.7 | 3.2 | 3.4 | 3.0 | 3.4 | 3.4 | 3.3 |  |

Notes:

1: Percent change in rate was calculated as “(mortality in 2013- mortality in 2006)/(mortality in 2006)×100”.

2: ^*^: *p*<0.05; ^**^: *p*<0.01
