# Supplementary material for: Trends in traumatic brain injury mortality in China, 2006–2013: A population-based longitudinal study
Source: PLoS Med. 2017 Jul 11;14(7):e1002332. doi: 10.1371/journal.pmed.1002332 (PMC5507407; doi:10.1371/journal.pmed.1002332)
Supplement: S4 Table — (DOCX) [file pmed.1002332.s006.docx]

**Supplementary 7 Table. Mortality rates from traumatic brain injury per 100,000 population (standard error) by 5-year age group in China, 2006-2013**

| **Age group** | **2006** | **2007** | **2008** | **2009** | **2010** | **2011** | **2012** | **2013** |
| --- | --- | --- | --- | --- | --- | --- | --- | --- |
| 0-4 years | 3.86 (0.29) | 4.14 (0.30) | 4.29 (0.30) | 3.80 (0.28) | 4.28 (0.30) | 5.01 (0.33) | 4.73 (0.33) | 4.71 (0.32) |
| 5-9 years | 3.11 (0.28) | 3.67 (0.30) | 4.06 (0.31) | 3.14 (0.27) | 3.36 (0.27) | 3.06 (0.27) | 2.79 (0.26) | 2.24 (0.22) |
| 10-14 years | 2.00 (0.19) | 2.68 (0.23) | 2.82 (0.24) | 2.57 (0.23) | 2.82 (0.25) | 2.77 (0.26) | 2.22 (0.23) | 2.22 (0.24) |
| 15-19 years | 5.97 (0.28) | 7.52 (0.32) | 7.30 (0.33) | 6.58 (0.32) | 7.13 (0.35) | 6.63 (0.33) | 5.98 (0.33) | 6.62 (0.36) |
| 20-24 years | 9.50 (0.40) | 11.76 (0.44) | 12.54 (0.44) | 11.15 (0.41) | 11.67 (0.41) | 11.01 (0.39) | 8.05 (0.32) | 6.84 (0.29) |
| 25-29 years | 11.22 (0.47) | 13.28 (0.51) | 13.48 (0.50) | 11.77 (0.46) | 11.87 (0.46) | 11.17 (0.42) | 10.67 (0.41) | 9.13 (0.38) |
| 30-34 years | 11.95 (0.43) | 13.89 (0.47) | 15.19 (0.51) | 12.97 (0.49) | 14.04 (0.52) | 11.93 (0.46) | 11.19 (0.44) | 9.61 (0.42) |
| 35-39 years | 14.77 (0.45) | 17.27 (0.49) | 18.55 (0.50) | 15.88 (0.47) | 14.73 (0.46) | 13.11 (0.42) | 12.03 (0.41) | 10.25 (0.39) |
| 40-44 years | 17.26 (0.52) | 17.88 (0.50) | 20.50 (0.53) | 18.85 (0.51) | 19.70 (0.52) | 18.34 (0.49) | 16.36 (0.46) | 15.08 (0.45) |
| 45-49 years | 14.86 (0.54) | 17.57 (0.61) | 22.12 (0.68) | 19.21 (0.60) | 21.10 (0.59) | 21.64 (0.58) | 18.96 (0.51) | 14.6 (0.42) |
| 50-54 years | 15.27 (0.53) | 17.73 (0.57) | 20.99 (0.61) | 16.54 (0.54) | 18.89 (0.59) | 16.33 (0.53) | 18.99 (0.62) | 18.83 (0.62) |
| 55-59 years | 16.25 (0.64) | 19.16 (0.68) | 21.50 (0.69) | 20.49 (0.65) | 22.55 (0.67) | 22.23 (0.64) | 22.13 (0.64) | 19.61 (0.59) |
| 60-64 years | 17.57 (0.81) | 20.94 (0.87) | 25.06 (0.92) | 23.89 (0.87) | 25.16 (0.87) | 26.16 (0.86) | 25.92 (0.83) | 23.58 (0.76) |
| 65-69 years | 19.47 (0.91) | 23.17 (1.00) | 26.27 (1.06) | 23.23 (1.00) | 27.94 (1.08) | 26.14 (0.99) | 28.34 (1.05) | 26.64 (1.00) |
| 70-74 years | 28.05 (1.24) | 29.11 (1.24) | 31.07 (1.26) | 28.69 (1.21) | 31.14 (1.27) | 27.34 (1.12) | 29.50 (1.19) | 27.97 (1.16) |
| 75-79 years | 34.66 (1.71) | 40.79 (1.83) | 39.56 (1.77) | 38.47 (1.71) | 40.15 (1.72) | 38.22 (1.58) | 39.43 (1.58) | 35.94 (1.47) |
| 80-84 years | 54.36 (2.99) | 61.00 (3.13) | 65.45 (3.17) | 64.16 (3.05) | 56.31 (2.83) | 58.59 (2.71) | 55.84 (2.51) | 48.06 (2.25) |
| 85 years and older | 138.48 (8.35) | 169.03 (9.28) | 195.44 (9.91) | 172.55 (9.28) | 183.31 (9.45) | 168.08 (8.21) | 89.15 (4.34) | 90.95 (4.23) |
