# Supplementary material for: Trends in traumatic brain injury mortality in China, 2006–2013: A population-based longitudinal study
Source: PLoS Med. 2017 Jul 11;14(7):e1002332. doi: 10.1371/journal.pmed.1002332 (PMC5507407; doi:10.1371/journal.pmed.1002332)
Supplement: S5 Table — (DOCX) [file pmed.1002332.s007.docx]

**Supplementary Table 5. Mortality rates from traumatic brain injury per 100,000 population (standard error) by cause and age group in China, 2006-2013**

| **Cause** | **Age group** | **2006** | **2007** | **2008** | **2009** | **2010** | **2011** | **2012** | **2013** | **% change in rate** |
| --- | --- | --- | --- | --- | --- | --- | --- | --- | --- | --- |
| **Motor vehicle crash** | 0-4 years | 1.39 (0.17) | 1.44 (0.18) | 1.80 (0.20) | 1.42 (0.17) | 1.84 (0.19) | 2.55 (0.24) | 2.44 (0.23) | 1.95 (0.21) | 40^*^ |
|  | 5-14 years | 1.01 (0.10) | 1.60 (0.13) | 1.28 (0.12) | 1.35 (0.12) | 1.67 (0.14) | 1.68 (0.14) | 1.38 (0.13) | 1.20 (0.12) | 20 |
|  | 15-24 years | 3.48 (0.16) | 4.63 (0.19) | 4.71 (0.19) | 4.91 (0.19) | 5.89 (0.21) | 5.42 (0.20) | 4.34 (0.18) | 3.95 (0.17) | 13^*^ |
|  | 25-44 years | 5.70 (0.15) | 6.93 (0.16) | 7.79 (0.17) | 7.64 (0.17) | 8.49 (0.18) | 7.87 (0.17) | 7.20 (0.16) | 6.18 (0.15) | 8^*^ |
|  | 45-64 years | 6.32 (0.19) | 8.00 (0.22) | 9.46 (0.23) | 9.18 (0.22) | 11.29 (0.24) | 11.46 (0.23) | 11.11 (0.23) | 9.39 (0.20) | 49^**^ |
|  | 65-74 years | 8.89 (0.46) | 10.10 (0.49) | 10.15 (0.49) | 11.44 (0.52) | 14.43 (0.58) | 13.88 (0.53) | 15.08 (0.57) | 13.95 (0.54) | 57^**^ |
|  | ≥75 years | 10.97 (0.74) | 16.20 (0.89) | 15.11 (0.85) | 15.35 (0.83) | 18.94 (0.91) | 17.78 (0.83) | 17.21 (0.76) | 13.96 (0.67) | 27^**^ |
| **Falls** | 0-4 years | 1.01 (0.15) | 1.20 (0.16) | 0.96 (0.14) | 0.81 (0.13) | 1.29 (0.16) | 1.25 (0.17) | 1.08 (0.16) | 1.29 (0.17) | 28 |
|  | 5-14 years | 0.39 (0.06) | 0.51 (0.07) | 0.52 (0.08) | 0.42 (0.07) | 0.43 (0.07) | 0.56 (0.08) | 0.56 (0.08) | 0.54 (0.08) | 39 |
|  | 15-24 years | 0.77 (0.08) | 0.96 (0.08) | 1.02 (0.09) | 1.04 (0.09) | 0.96 (0.09) | 1.04 (0.09) | 1.00 (0.09) | 0.88 (0.08) | 14 |
|  | 25-44 years | 1.84 (0.08) | 2.13 (0.09) | 2.45 (0.10) | 2.16 (0.09) | 2.24 (0.09) | 2.28 (0.09) | 2.04 (0.09) | 1.98 (0.09) | 8 |
|  | 45-64 years | 2.95 (0.13) | 3.51 (0.14) | 4.27 (0.15) | 3.80 (0.14) | 4.26 (0.15) | 4.06 (0.14) | 4.38 (0.14) | 4.30 (0.14) | 46^**^ |
|  | 65-74 years | 7.07 (0.41) | 7.22 (0.41) | 8.31 (0.44) | 6.89 (0.40) | 7.38 (0.41) | 6.58 (0.37) | 7.36 (0.40) | 7.43 (0.40) | 5 |
|  | ≥75 years | 29.04 (1.21) | 30.88 (1.23) | 33.16 (1.26) | 32.51 (1.21) | 30.10 (1.15) | 29.99 (1.08) | 27.08 (0.96) | 27.51 (0.94) | -5 |
| **Struck by/against** | 0-4 years | 0.13 (0.05) | 0.26 (0.07) | 0.23 (0.07) | 0.17 (0.06) | 0.18 (0.06) | 0.16 (0.06) | 0.16 (0.06) | 0.13 (0.05) | 3 |
|  | 5-14 years | 0.11 (0.03) | 0.13 (0.04) | 0.17 (0.04) | 0.09 (0.04) | 0.14 (0.04) | 0.11 (0.04) | 0.11 (0.04) | 0.09 (0.03) | -14 |
|  | 15-24 years | 0.30 (0.05) | 0.41 (0.06) | 0.54 (0.06) | 0.35 (0.06) | 0.42 (0.06) | 0.43 (0.06) | 0.25 (0.04) | 0.33 (0.05) | 10 |
|  | 25-44 years | 0.98 (0.06) | 0.99 (0.06) | 1.13 (0.07) | 0.94 (0.06) | 0.97 (0.06) | 0.71 (0.05) | 0.72 (0.05) | 0.59 (0.05) | -40^**^ |
|  | 45-64 years | 0.90 (0.07) | 0.95 (0.07) | 1.23 (0.08) | 0.92 (0.07) | 1.01 (0.07) | 0.88 (0.06) | 0.94 (0.07) | 0.90 (0.06) | 0 |
|  | 65-74 years | 0.70 (0.13) | 0.71 (0.13) | 0.84 (0.14) | 0.58 (0.11) | 0.51 (0.11) | 0.60 (0.11) | 0.79 (0.13) | 0.65 (0.12) | -7 |
|  | ≥75 years | 1.15 (0.24) | 0.39 (0.14) | 1.52 (0.27) | 0.91 (0.20) | 0.93 (0.20) | 0.86 (0.18) | 0.95 (0.18) | 0.71 (0.15) | -39 |
| **All others** | 0-4 years | 1.33 (0.17) | 1.24 (0.16) | 1.29 (0.17) | 1.40 (0.17) | 0.96 (0.14) | 1.05 (0.15) | 1.05 (0.15) | 1.33 (0.17) | 0 |
|  | 5-14 years | 0.96 (0.10) | 0.88 (0.10) | 1.42 (0.12) | 0.98 (0.10) | 0.85 (0.10) | 0.56 (0.08) | 0.46 (0.07) | 0.39 (0.07) | -59^**^ |
|  | 15-24 years | 2.98 (0.15) | 3.48 (0.16) | 3.58 (0.16) | 2.60 (0.14) | 2.33 (0.13) | 2.11 (0.13) | 1.61 (0.11) | 1.59 (0.11) | -47^**^ |
|  | 25-44 years | 5.45 (0.15) | 5.79 (0.15) | 5.90 (0.15) | 4.44 (0.13) | 3.66 (0.12) | 3.02 (0.11) | 2.80 (0.10) | 2.48 (0.10) | -55^**^ |
|  | 45-64 years | 5.57 (0.18) | 6.10 (0.19) | 7.13 (0.20) | 5.60 (0.17) | 5.00 (0.16) | 4.70 (0.15) | 4.55 (0.15) | 3.76 (0.13) | -33^**^ |
|  | 65-74 years | 6.57 (0.40) | 7.81 (0.43) | 9.15 (0.46) | 6.82 (0.40) | 7.05 (0.40) | 5.61 (0.34) | 5.62 (0.35) | 5.19 (0.33) | -21^**^ |
|  | ≥75 years | 9.81 (0.70) | 11.88 (0.76) | 12.5 (0.77) | 9.99 (0.67) | 8.17 (0.60) | 8.52 (0.58) | 7.13 (0.49) | 6.42 (0.45) | -35^**^ |

Notes:

1: Percent change in rate was calculated as “(mortality in 2013- mortality in 2006)/(mortality in 2006)×100”.

2: ^*^: *p*<0.05; ^**^: *p*<0.01.
