# Supplementary material for: Trends in traumatic brain injury mortality in China, 2006–2013: A population-based longitudinal study
Source: PLoS Med. 2017 Jul 11;14(7):e1002332. doi: 10.1371/journal.pmed.1002332 (PMC5507407; doi:10.1371/journal.pmed.1002332)
Supplement: S6 Table — (DOCX) [file pmed.1002332.s008.docx]

**Supplementary Table 6. Age-standardized mortality rates from traumatic brain injury due to motor vehicle crashes per 100,000 population (standard error) by road user and location in China, 2006-2013**

| **Cause** | **Location** | **2006** | **2007** | **2008** | **2009** | **2010** | **2011** | **2012** | **2013** | **% change in rate** |
| --- | --- | --- | --- | --- | --- | --- | --- | --- | --- | --- |
| **Occupant** | Urban | 0.47 (0.04) | 0.65 (0.05) | 0.84 (0.05) | 0.83 (0.05) | 0.76 (0.05) | 0.60 (0.04) | 0.55 (0.04) | 0.46 (0.04) | -2 |
|  | Rural | 0.88 (0.04) | 1.05 (0.05) | 1.33 (0.05) | 1.12 (0.05) | 1.29 (0.05) | 1.59 (0.06) | 1.56 (0.06) | 1.43 (0.06) | 63^**^ |
|  | Ratio | 1.9 | 1.6 | 1.6 | 1.3 | 1.7 | 2.5 | 2.8 | 3.0 |  |
| **Motorcyclist** | Urban | 0.52 (0.04) | 0.71 (0.05) | 0.8 (0.05) | 0.95 (0.06) | 1.09 (0.06) | 0.94 (0.05) | 0.68 (0.04) | 0.69 (0.04) | 33^**^ |
|  | Rural | 1.70 (0.06) | 2.05 (0.07) | 2.39 (0.07) | 2.48 (0.07) | 2.94 (0.08) | 3.20 (0.08) | 3.02 (0.08) | 2.61 (0.08) | 54^**^ |
|  | Ratio | 3.3 | 2.9 | 3.0 | 2.6 | 2.7 | 3.3 | 4.4 | 3.8 |  |
| **Pedal cyclist** | Urban | 0.29 (0.03) | 0.45 (0.04) | 0.43 (0.04) | 0.41 (0.04) | 0.41 (0.04) | 0.36 (0.03) | 0.44 (0.04) | 0.27 (0.04) | -7 |
|  | Rural | 0.44 (0.03) | 0.55 (0.03) | 0.76 (0.04) | 0.70 (0.04) | 0.87 (0.04) | 0.99 (0.05) | 0.97 (0.05) | 0.84 (0.05) | 91^**^ |
|  | Ratio | 1.5 | 1.2 | 1.8 | 1.7 | 2.1 | 2.8 | 2.2 | 3.0 |  |
| **Pedestrian** | Urban | 1.46 (0.07) | 1.84 (0.08) | 2.43 (0.09) | 2.36 (0.09) | 2.40 (0.09) | 2.36 (0.08) | 2.19 (0.08) | 1.90 (0.08) | 30^**^ |
|  | Rural | 2.64 (0.07) | 3.62 (0.09) | 3.16 (0.08) | 3.52 (0.09) | 4.56 (0.10) | 4.51 (0.10) | 4.17 (0.09) | 3.60 (0.09) | 36^**^ |
|  | Ratio | 1.8 | 2.0 | 1.3 | 1.5 | 1.9 | 1.9 | 1.9 | 1.8 |  |
| **All others** | Urban | 0.28 (0.03) | 0.40 (0.04) | 0.40 (0.04) | 0.42 (0.04) | 0.75 (0.05) | 0.52 (0.04) | 0.45 (0.04) | 0.26 (0.04) | -7 |
|  | Rural | 0.67 (0.04) | 0.59 (0.03) | 0.71 (0.04) | 0.48 (0.03) | 0.52 (0.03) | 0.45 (0.03) | 0.40 (0.03) | 0.37 (0.03) | -45^**^ |
|  | Ratio | 2.4 | 1.5 | 1.8 | 1.1 | 0.7 | 0.8 | 0.9 | 1.4 |  |

Notes:

1: Percent change in rate was calculated as “(mortality in 2013- mortality in 2006)/(mortality in 2006)×100”.

2: ^*^: *p*<0.05; ^**^: *p*<0.01.
