# Supplementary material for: Trends in traumatic brain injury mortality in China, 2006–2013: A population-based longitudinal study
Source: PLoS Med. 2017 Jul 11;14(7):e1002332. doi: 10.1371/journal.pmed.1002332 (PMC5507407; doi:10.1371/journal.pmed.1002332)
Supplement: S7 Table — (DOCX) [file pmed.1002332.s009.docx]

**Supplementary Table 7. Age-standardized mortality rates from traumatic brain injury due to motor vehicle crashes per 100,000 population (standard error) by road user and sex in China, 2006-2013**

| **Cause** | **Sex** | **2006** | **2007** | **2008** | **2009** | **2010** | **2011** | **2012** | **2013** | **% change in rate** |
| --- | --- | --- | --- | --- | --- | --- | --- | --- | --- | --- |
| **Occupant** | Male | 1.15 (0.05) | 1.45 (0.06) | 1.77 (0.07) | 1.62 (0.06) | 1.65 (0.06) | 1.76 (0.07) | 1.76 (0.06) | 1.49 (0.06) | 30^**^ |
|  | Female | 0.30 (0.03) | 0.33 (0.03) | 0.49 (0.04) | 0.37 (0.03) | 0.50 (0.04) | 0.50 (0.04) | 0.44 (0.03) | 0.49 (0.03) | 63^**^ |
|  | Ratio | 3.8 | 4.5 | 3.6 | 4.4 | 3.3 | 3.5 | 4.0 | 3.1 |  |
| **Motorcyclist** | Male | 2.16 (0.08) | 2.59 (0.08) | 2.98 (0.09) | 3.18 (0.09) | 3.78 (0.10) | 3.66 (0.09) | 3.29 (0.09) | 2.91 (0.09) | 35^**^ |
|  | Female | 0.33 (0.03) | 0.45 (0.03) | 0.52 (0.04) | 0.53 (0.04) | 0.61 (0.04) | 0.62 (0.04) | 0.60 (0.04) | 0.54 (0.04) | 64^**^ |
|  | Ratio | 6.5 | 5.8 | 5.7 | 5.9 | 6.2 | 5.9 | 5.5 | 5.4 |  |
| **Pedal cyclist** | Male | 0.56 (0.04) | 0.70 (0.04) | 0.86 (0.05) | 0.85 (0.05) | 0.97 (0.05) | 0.93 (0.05) | 1.04 (0.05) | 0.79 (0.05) | 41^**^ |
|  | Female | 0.20 (0.02) | 0.31 (0.03) | 0.40 (0.03) | 0.33 (0.03) | 0.41 (0.03) | 0.48 (0.03) | 0.45 (0.03) | 0.40 (0.03) | 100^**^ |
|  | Ratio | 2.7 | 2.2 | 2.2 | 2.6 | 2.4 | 1.9 | 2.3 | 2.0 |  |
| **Pedestrian** | Male | 2.97 (0.09) | 4.07 (0.10) | 4.05 (0.10) | 4.25 (0.10) | 5.16 (0.11) | 5.13 (0.11) | 4.61 (0.11) | 4.05 (0.11) | 36^**^ |
|  | Female | 1.39 (0.06) | 1.75 (0.07) | 1.66 (0.07) | 1.84 (0.07) | 2.21 (0.08) | 1.94 (0.07) | 2.00 (0.07) | 1.64 (0.07) | 18^**^ |
|  | Ratio | 2.1 | 2.3 | 2.4 | 2.3 | 2.3 | 2.6 | 2.3 | 2.5 |  |
| **All others** | Male | 0.81 (0.05) | 0.78 (0.04) | 0.91 (0.05) | 0.72 (0.04) | 0.87 (0.05) | 0.71 (0.04) | 0.65 (0.04) | 0.47 (0.04) | -42^**^ |
|  | Female | 0.23 (0.02) | 0.25 (0.03) | 0.27 (0.03) | 0.18 (0.02) | 0.32 (0.03) | 0.23 (0.02) | 0.18 (0.02) | 0.16 (0.02) | -30^*^ |
|  | Ratio | 3.5 | 3.0 | 3.4 | 3.9 | 2.7 | 3.1 | 3.7 | 2.9 |  |

Notes:

1: Percent change in rate was calculated as “(mortality in 2013- mortality in 2006)/(mortality in 2006)×100”.

2: ^*^: *p*<0.05; ^**^: *p*<0.01.
