# Supplementary material for: Trends in traumatic brain injury mortality in China, 2006–2013: A population-based longitudinal study
Source: PLoS Med. 2017 Jul 11;14(7):e1002332. doi: 10.1371/journal.pmed.1002332 (PMC5507407; doi:10.1371/journal.pmed.1002332)
Supplement: S8 Table — (DOCX) [file pmed.1002332.s010.docx]

**Supplementary Table 8. Mortality rates from traumatic brain injury due to motor vehicle crashes per 100,000 population (standard error) by road user and age group in China, 2006-2013**

| **Road User** | **Age group** | **2006** | **2007** | **2008** | **2009** | **2010** | **2011** | **2012** | **2013** | **% change in rate** |
| --- | --- | --- | --- | --- | --- | --- | --- | --- | --- | --- |
| **Occupant** | All ages | 0.72 (0.03) | 0.89 (0.03) | 1.14 (0.04) | 1.00 (0.04) | 1.08 (0.04) | 1.14 (0.04) | 1.11 (0.04) | 0.99 (0.03) | 38^**^ |
|  | 0-4 years | 0.13 (0.05) | 0.15 (0.06) | 0.40 (0.09) | 0.23 (0.07) | 0.35 (0.08) | 0.27 (0.08) | 0.38 (0.09) | 0.33 (0.09) | 159^*^ |
|  | 5-14 years | 0.16 (0.04) | 0.10 (0.03) | 0.21 (0.05) | 0.14 (0.04) | 0.14 (0.04) | 0.23 (0.05) | 0.22 (0.05) | 0.14 (0.04) | -11 |
|  | 15-24 years | 0.49 (0.06) | 0.71 (0.07) | 0.72 (0.07) | 0.64 (0.07) | 0.74 (0.08) | 0.74 (0.07) | 0.66 (0.07) | 0.48 (0.06) | -2 |
|  | 25-44 years | 1.01 (0.06) | 1.23 (0.07) | 1.55 (0.08) | 1.34 (0.07) | 1.46 (0.08) | 1.08 (0.06) | 1.44 (0.07) | 1.36 (0.07) | 34^**^ |
|  | 45-64 years | 0.93 (0.07) | 1.03 (0.08) | 1.42 (0.09) | 1.45 (0.09) | 1.40 (0.08) | 1.15 (0.07) | 1.48 (0.08) | 1.27 (0.07) | 37^**^ |
|  | 65-74 years | 0.60 (0.12) | 1.02 (0.16) | 0.95 (0.15) | 0.74 (0.13) | 1.22 (0.17) | 0.70 (0.12) | 1.35 (0.17) | 1.60 (0.18) | 166^**^ |
|  | ≥75 years | 0.60 (0.17) | 1.28 (0.25) | 1.76 (0.29) | 0.86 (0.20) | 0.93 (0.20) | 0.74 (0.17) | 1.19 (0.20) | 0.87 (0.17) | 44 |
| **Motorcyclist** | All ages | 1.25 (0.04) | 1.54 (0.04) | 1.77 (0.05) | 1.88 (0.05) | 2.23 (0.05) | 2.17 (0.05) | 1.97 (0.05) | 1.74 (0.05) | 39^**^ |
|  | 0-4 years | 0.09 (0.04) | 0.13 (0.05) | 0.11 (0.05) | 0.15 (0.06) | 0.08 (0.04) | 0.25 (0.07) | 0.18 (0.06) | 0.13 (0.05) | 56 |
|  | 5-14 years | 0.15 (0.04) | 0.15 (0.04) | 0.17 (0.04) | 0.17 (0.04) | 0.17 (0.04) | 0.21 (0.05) | 0.18 (0.05) | 0.19 (0.05) | 27 |
|  | 15-24 years | 1.54 (0.11) | 1.72 (0.11) | 1.83 (0.12) | 1.98 (0.12) | 2.51 (0.14) | 1.74 (0.11) | 1.77 (0.12) | 1.77 (0.12) | 15 |
|  | 25-44 years | 1.88 (0.09) | 2.36 (0.10) | 2.67 (0.10) | 2.67 (0.10) | 3.06 (0.11) | 2.18 (0.09) | 2.45 (0.10) | 2.02 (0.09) | 8 |
|  | 45-64 years | 1.22 (0.08) | 1.52 (0.09) | 2.01 (0.11) | 2.21 (0.11) | 2.73 (0.12) | 2.45 (0.11) | 2.84 (0.12) | 2.56 (0.11) | 111^**^ |
|  | 65-74 years | 0.72 (0.13) | 0.80 (0.14) | 0.75 (0.13) | 1.23 (0.17) | 1.27 (0.17) | 1.28 (0.16) | 1.74 (0.19) | 1.60 (0.18) | 121^**^ |
|  | ≥75 years | 0.25 (0.11) | 0.74 (0.19) | 0.71 (0.18) | 0.86 (0.20) | 0.79 (0.19) | 0.62 (0.16) | 1.09 (0.19) | 0.90 (0.17) | 259^**^ |
| **Pedal cyclist** | All ages | 0.38 (0.02) | 0.5 (0.03) | 0.63 (0.03) | 0.58 (0.03) | 0.69 (0.03) | 0.7 (0.03) | 0.74 (0.03) | 0.59 (0.03) | 55^**^ |
|  | 0-4 years | 0.02 (0.02) | 0.02 (0.02) | 0.00 (0.00) | 0.06 (0.04) | 0.06 (0.04) | 0.13 (0.05) | 0.04 (0.03) | 0.16 (0.06) | 626^*^ |
|  | 5-14 years | 0.10 (0.03) | 0.23 (0.05) | 0.12 (0.04) | 0.13 (0.04) | 0.14 (0.04) | 0.11 (0.04) | 0.13 (0.04) | 0.07 (0.03) | -26 |
|  | 15-24 years | 0.13 (0.03) | 0.24 (0.04) | 0.26 (0.04) | 0.28 (0.05) | 0.26 (0.05) | 0.23 (0.04) | 0.25 (0.04) | 0.19 (0.04) | 43 |
|  | 25-44 years | 0.32 (0.04) | 0.32 (0.03) | 0.47 (0.04) | 0.43 (0.04) | 0.46 (0.04) | 0.32 (0.03) | 0.45 (0.04) | 0.34 (0.04) | 8 |
|  | 45-64 years | 0.65 (0.06) | 0.94 (0.07) | 1.16 (0.08) | 0.95 (0.07) | 1.28 (0.08) | 0.99 (0.07) | 1.39 (0.08) | 1.07 (0.07) | 66^**^ |
|  | 65-74 years | 1.13 (0.17) | 1.11 (0.16) | 1.47 (0.18) | 1.65 (0.20) | 1.82 (0.20) | 1.67 (0.19) | 2.10 (0.21) | 1.90 (0.20) | 67^**^ |
|  | ≥75 years | 0.55 (0.17) | 1.28 (0.25) | 1.47 (0.26) | 1.59 (0.27) | 1.68 (0.27) | 1.32 (0.23) | 2.27 (0.28) | 1.73 (0.24) | 215^**^ |
| **Pedestrian** | All ages | 2.17 (0.05) | 2.92 (0.06) | 2.86 (0.06) | 3.05 (0.06) | 3.68 (0.07) | 3.53 (0.07) | 3.3 (0.06) | 2.84 (0.06) | 31^**^ |
|  | 0-4 years | 1.03 (0.15) | 0.97 (0.14) | 1.12 (0.15) | 0.84 (0.13) | 1.17 (0.15) | 1.23 (0.17) | 1.70 (0.20) | 1.29 (0.17) | 25 |
|  | 5-14 years | 0.48 (0.07) | 1.02 (0.11) | 0.68 (0.09) | 0.77 (0.09) | 1.07 (0.11) | 0.57 (0.08) | 0.74 (0.09) | 0.72 (0.09) | 51^*^ |
|  | 15-24 years | 0.85 (0.08) | 1.52 (0.11) | 1.52 (0.11) | 1.63 (0.11) | 1.82 (0.12) | 1.26 (0.10) | 1.39 (0.10) | 1.28 (0.10) | 50^**^ |
|  | 25-44 years | 1.91 (0.09) | 2.41 (0.10) | 2.41 (0.10) | 2.69 (0.10) | 2.90 (0.11) | 1.89 (0.08) | 2.46 (0.10) | 2.06 (0.09) | 8 |
|  | 45-64 years | 2.82 (0.13) | 3.88 (0.15) | 4.05 (0.15) | 3.97 (0.14) | 5.09 (0.16) | 3.58 (0.13) | 4.79 (0.15) | 4.13 (0.13) | 46^**^ |
|  | 65-74 years | 5.67 (0.37) | 6.34 (0.39) | 5.91 (0.37) | 7.33 (0.41) | 8.99 (0.46) | 5.84 (0.35) | 9.08 (0.44) | 8.34 (0.42) | 47^**^ |
|  | ≥75 years | 8.36 (0.65) | 11.88 (0.76) | 10.36 (0.70) | 11.12 (0.71) | 14.61 (0.80) | 9.84 (0.62) | 11.91 (0.64) | 9.63 (0.56) | 15 |
| **All others** | All ages | 0.52 (0.03) | 0.52 (0.03) | 0.59 (0.03) | 0.46 (0.02) | 0.6 (0.03) | 0.47 (0.02) | 0.41 (0.02) | 0.32 (0.02) | -38^**^ |
|  | 0-4 years | 0.09 (0.04) | 0.17 (0.06) | 0.17 (0.06) | 0.15 (0.06) | 0.18 (0.06) | 0.04 (0.03) | 0.13 (0.05) | 0.02 (0.02) | -74 |
|  | 5-14 years | 0.12 (0.04) | 0.09 (0.03) | 0.10 (0.03) | 0.14 (0.04) | 0.14 (0.04) | 0.07 (0.03) | 0.11 (0.04) | 0.08 (0.03) | -29 |
|  | 15-24 years | 0.45 (0.06) | 0.42 (0.06) | 0.38 (0.05) | 0.39 (0.05) | 0.56 (0.07) | 0.19 (0.04) | 0.28 (0.05) | 0.23 (0.04) | -49^**^ |
|  | 25-44 years | 0.55 (0.05) | 0.61 (0.05) | 0.69 (0.05) | 0.51 (0.04) | 0.60 (0.05) | 0.30 (0.03) | 0.39 (0.04) | 0.39 (0.04) | -30^**^ |
|  | 45-64 years | 0.68 (0.06) | 0.62 (0.06) | 0.80 (0.07) | 0.60 (0.06) | 0.78 (0.06) | 0.27 (0.04) | 0.61 (0.05) | 0.36 (0.04) | -47^**^ |
|  | 65-74 years | 0.68 (0.13) | 0.80 (0.14) | 1.02 (0.15) | 0.47 (0.10) | 1.13 (0.16) | 0.49 (0.10) | 0.82 (0.13) | 0.46 (0.10) | -31 |
|  | ≥75 years | 1.15 (0.24) | 0.88 (0.21) | 0.76 (0.19) | 0.86 (0.20) | 0.88 (0.20) | 0.27 (0.10) | 0.75 (0.16) | 0.80 (0.16) | -30 |

Notes:

1: Percent change in rate was calculated as “(mortality in 2013- mortality in 2006)/(mortality in 2006)×100”.

2: ^*^: *p*<0.05; ^**^: *p*<0.01.
